# Supplementary material for: Short-term availability of adult-born neurons for memory encoding
Source: Nat Commun. 2019 Dec 6;10:5609. doi: 10.1038/s41467-019-13521-7 (PMC6897887; doi:10.1038/s41467-019-13521-7)
Supplement: Supplementary file 1 — Supplementary Information [file 41467_2019_13521_MOESM1_ESM.pdf]

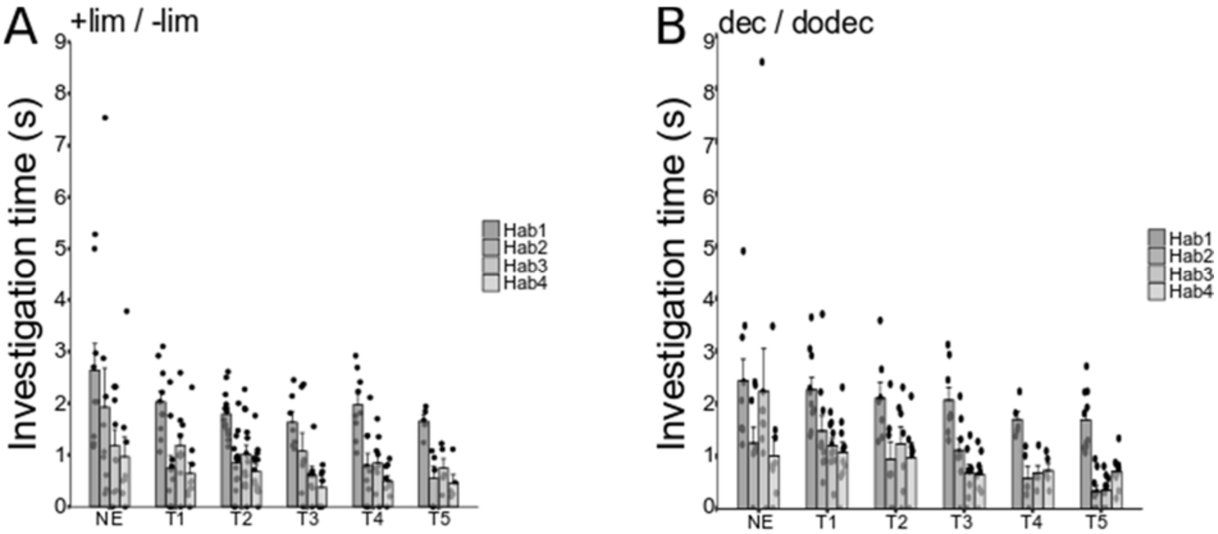

Supplementary Figure 1. Habituation behavior during habituation/dishabituation test.
A. Habituation (Hab1-Hab4) of the habituation/dishabituation task for the pair +lim/-lim. All groups show habituation with repeated exposure (T1 n=11; T2 n=15; T3 n=10; T4 n=9; T5 n=5). **B.** Habituation (Hab1-Hab4) of the habituation/dishabituation task for the pair dec/dodec (NE n=9; T1 n=10; T2 n=7; T3 n=8; T4 n=5; T5 n=10). All groups show habituation with repeated exposure. Data are represented as data points and mean ± sem.

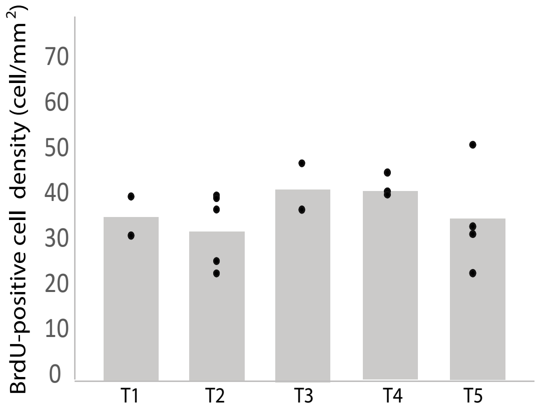

Supplementary Figure 2. Density of BrdU-positive cells in the non-enriched groups. No effect of time was observed on the density of BrdU-positive cells in the non-enriched groups (n=2-4/group). Data are represented as data points, grey bars give the mean.

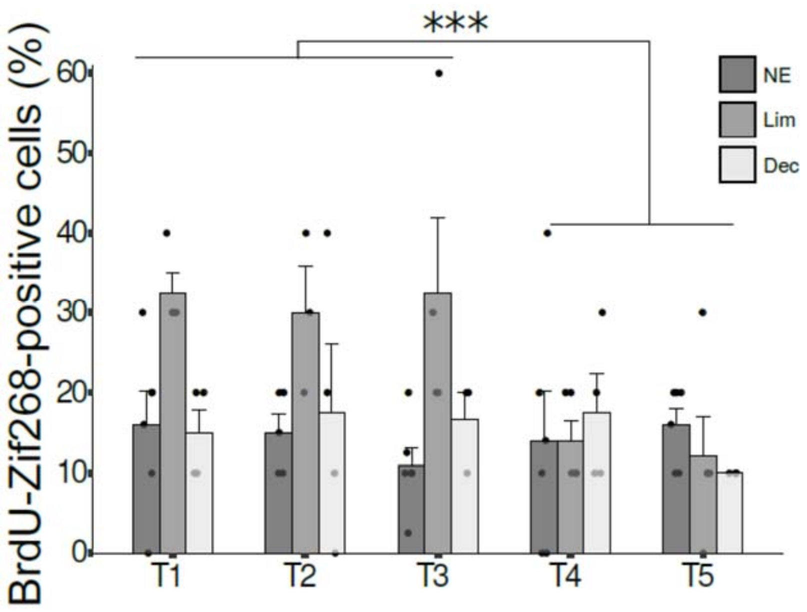

Supplementary Figure 3. Percentage of BrdU cells co-expressing Zif268 in the different experimental groups (Non-enriched group in response to +lim (NE; n=5-6/group); +lim/-lim enriched in response to +lim (lim; n=3-5) and +lim/-lim enriched in response to dec (dec; n=3-4)). A 2-way ANOVA showed a group effect ($F_{(2,54)}$=6.54, p=0.002). Interestingly, the percentage of Zif268 expression in adult-born neurons in response to +lim evolved with time ($F_{(4,16)}$=3.85, p=0.02) with a higher percentage in enriched animals at T1-T3 compared to T4-T5 (p=0.00022). This result is in accordance with the performance of discrimination and specific to the learned odorant (no time effect for response to dec, $F_{(4,14)}$=0.41, p=079). Regarding the non-enriched group, no time effect was observed ($F_{(4,24)}$=0.32, p=0.85) (n=4-6/group). Data are represented as data points and mean ± sem.

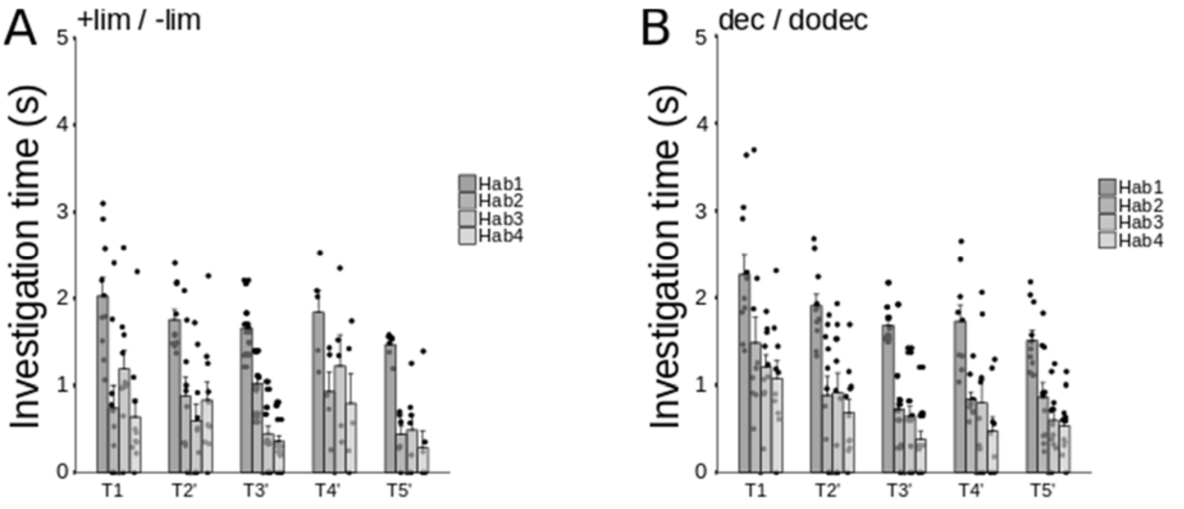

Supplementary Figure 4. Habituation behavior during habituation/dishabituation test. **A.** Habituation (Hab1-Hab4) of the habituation/dishabituation task for +lim/-lim. All groups show habituation with repeated odorant exposure (T1 n=11; T2’ n=11; T3’ n=11; T4’ n=6; T5’ n=7). **B.** Habituation (Hab1-Hab4) of the habituation/dishabituation task for dec/dodec (T1 n=10; T2’ n=11; T3’ n=11; T4’ n=10; T5’ n=11). All groups show habituation with repeated exposure. Data are represented as data points and mean ± sem.

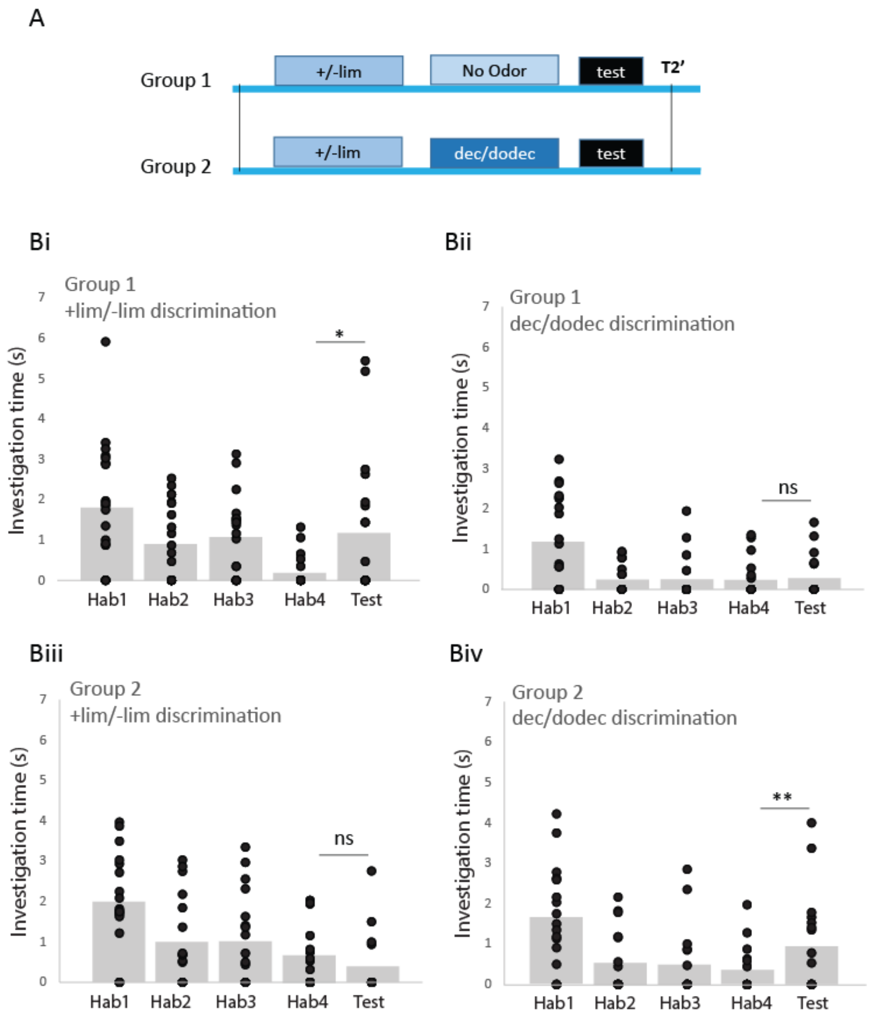

Supplementary Figure 5. A. Experimental design using the mouse strain 129. The interval between the enrichment with +lim/-lim and dec/dodec (Group 2) or between the enrichment with +lim/-lim and no enrichment (Group 1) is of 4 days (T2'). **B.** Behavioral results. Animals significantly habituate in all experimental groups (group1, +lim/-lim $F_{(3,57)}=9.8$ $p<0.0001$; dec/dodec $F_{(3,57)}=10.21$ $p<0.0001$; group 2, +lim/-lim $F_{(3,51)}=6.12$ $p=0.0012$; dec/dodec $F_{(3,51)}=9.03$ $p<0.0001$) with Hab1 significantly superior to Hab4 ($p<0.005$). Animals of Group 1 discriminate **(Bi)** +lim from –lim (n=20) but not **(Bii)** dec/dodec (n=20)**.** In Group 2, animals do not discriminate **(Biii)** +lim from –lim (n=19) but do discriminate **(Biv)** dec/dodec (n=19)**.** *p<0.05; **p<0.01. Single data points are represented, grey bars give the mean.

.

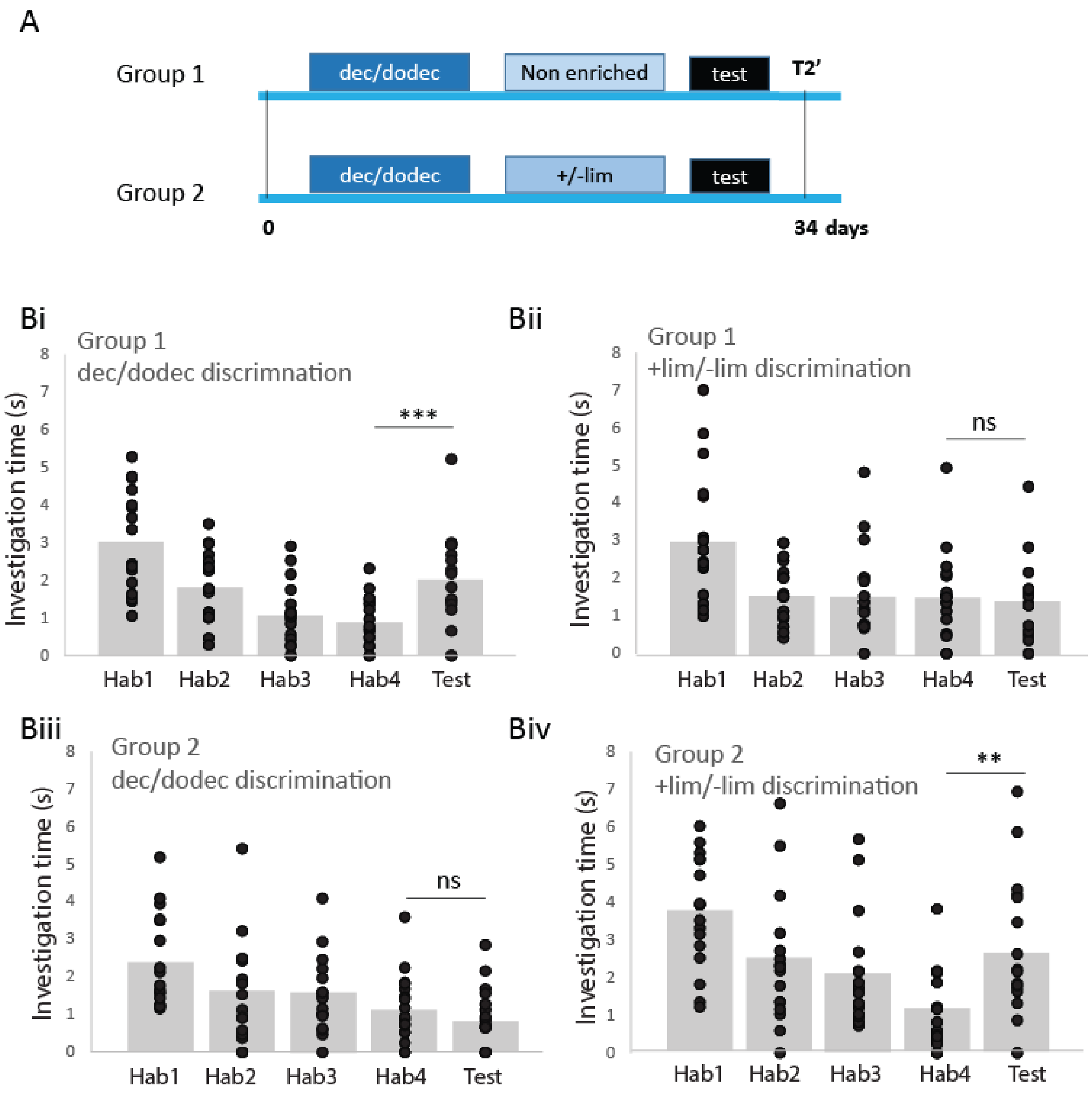

Supplementary Figure 6. A. Experimental design of inverted enrichments. The interval between the enrichment with dec/dodec and +lim/-lim (Group 2) or between the enrichment with dec/dodec and no enrichment (Group 1) is of 4 days (T2'). **B.** Behavioral results. Animals significantly habituate in all experimental groups (group 1, dec/dodec $F_{(3,48)}=25.88$ $p<0.0001$; +lim/-lim $F_{(3,48)}=11.07$ $p<0.0001$; group 2, dec/dodec $F_{(3,48)}=7$ $p=0.0005$; +lim/-lim $F_{(3,48)}=19.21$ $p<0.0001$) with Hab1 significantly superior to Hab4 ($p<0.005$). In group 1, animals discriminate **(Bi)** dec from dodec (n=17) but not **(Biv)** +lim/-lim (n=17). In group 2, animals do not discriminate **(Biii)** dec from dodec (n=17) but do discriminate (**Biv)** +lim/-lim (n=17). **p<0.01 ; ***p<0.00. Single data points are represented, grey bars give the mean.

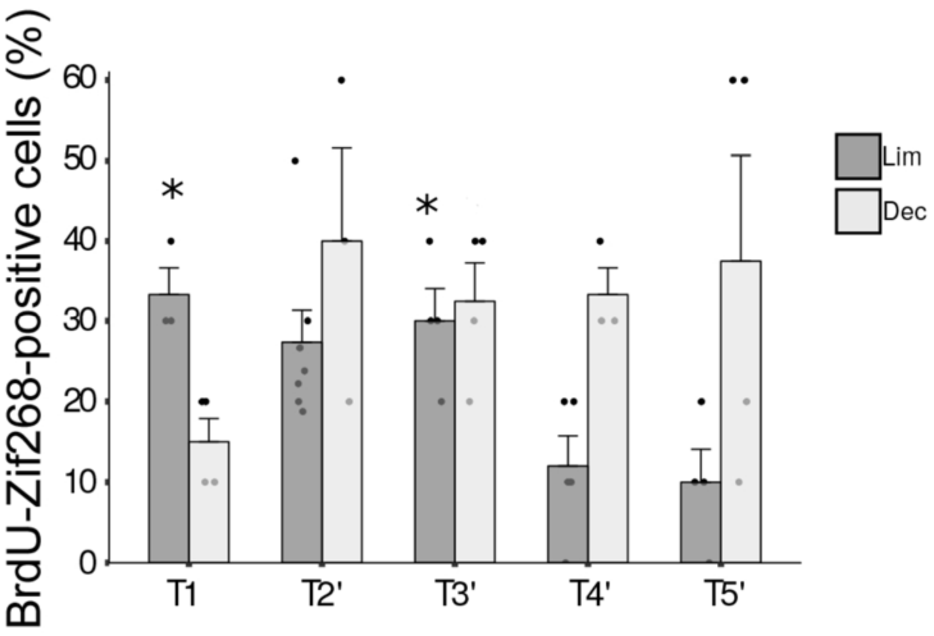

Supplementary Figure 7. Percentage of BrdU cells co-expressing Zif268 in the different experimental groups (+lim/-lim enriched in response to +lim (Lim; n=3-7) and +lim/-lim enriched in response to dec (dec; n=3-4/group)). The responses to +lim and dec were different ($F_{(1,31)}$=5.24, p=0.02). In response to +lim, the percentage of BrdU/Zif268-positive cells evolved with the time ($F_{(4,18)}$=6.01, p<0.002). It is higher in groups that discriminate +lim from –lim (T1 and T3') than in groups that do not discriminate (T2', T4' and T5') (p=0.007). In enriched animals in response to dec, the percentage of BrdU/Zif268-positive cells did not evolved significantly with time ($F_{(4,13)}$=1.43, p=0.27).

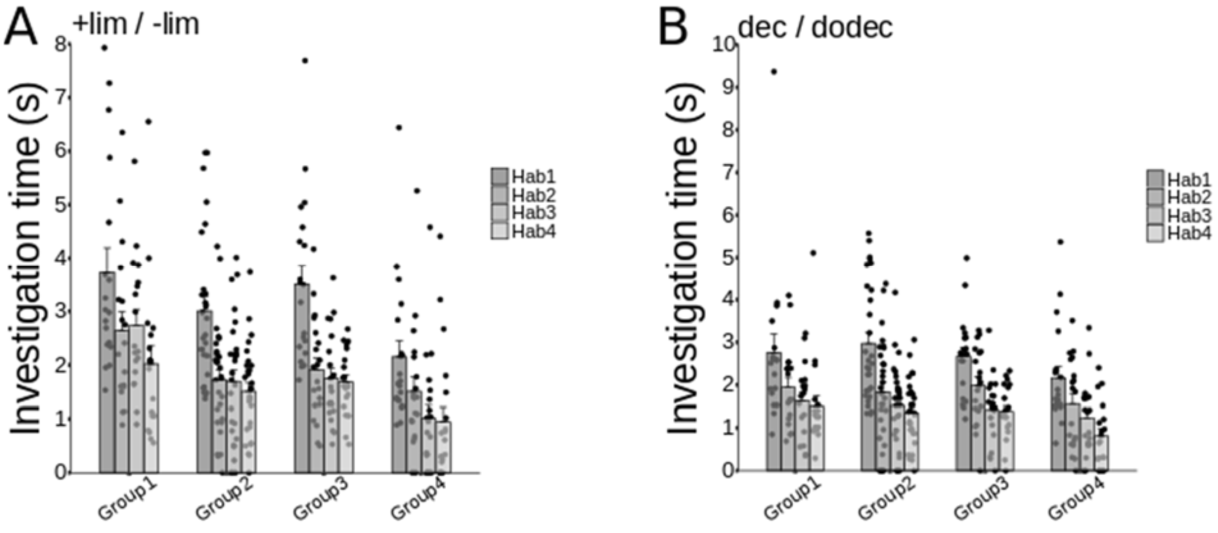

Supplementary Figure 8. Habituation behavior during habituation/dishabituation test. **A.** Habituation (Hab1-Hab4) of the habituation/dishabituation task for the pair +lim/-lim. All groups show habituation with repeated exposure (Group 1 n=18; Group 2 n=18; Group 3 n=20; Group 4 n=10). **B.** Habituation (Hab1-Hab4) of the habituation/dishabituation task for the pair dec/dodec (Group 1 n=18; Group 2 n=18; Group 3 n=20; Group 4 n=10). All groups show habituation with repeated exposure. Data are represented as data points and mean ± sem.

Group	Odorants	Habituation			Discrimination		
		Repeated measure ANOVA		Significant	T-test (1 tail, paired)		Significant
T1NE	+lim/-lim	F(3,24)=3.75	p = 0.024	*	T = -0.74, df=8	P=0.24	**ns**
	dec/dodec	F(3,24)=4.15	P=0.017	*	T=-1.49, df=8	P=0.09	**ns**
T1	+lim/-lim	F(3,33)=7.77	P=0.0005	***	T=-2.43, df=10	P=0.018	*
	dec/dodec	F(3,30)=6.89	P=0.001	***	T=1.24, df=9	P=0.88	**ns**
T2	+lim/-lim	F(3,48)=7.74	P<0.0003	***	T=-2.36, df=14	P=0.017	*
	dec/dodec	F(3,19)=5.27	P=0.008	***	T=0.69, df=6	P=0.74	**ns**
T3	+lim/-lim	F(3,29)=10.36	P=0.0001	***	T=-1.87, df=9	P=0.047	*
	dec/dodec	F(3,22)=11.03	P=0.0004	***	T=-1.45, df=7	P=0.095	**ns**
T4	+lim/-lim	F(3,25)=20.21	P<0.0001	***	T=-1.12, df=8	P=0.15	**ns**
	dec/dodec	F(3,13)=21.32	P<0.0001	***	T=0.74, df=4	P=0.75	**ns**
T5	+lim/-lim	F(3,12)=16.88	P=0.0001	***	T=-0.99, df=4	P=0.19	**ns**
	dec/dodec	F(3,28)=31.5	P<0.0001	***	T=1.02, df=9	P=0.83	**ns**

Supplementary Table 1. Statistical results

Group	Odorant	Habituation			Discrimination		
		Repeated measure ANOVA		Significant	T-test (1 tail, paired)		Significant
T1	+lim/-lim	F(3,29)=11.82	P<0.0001	***	T=-3.3, df=9	P=0.005	**
	dec/dodec	F(3,30)=6.89	P=0.001	***	T=1.24, df=9	P=0.88	**ns**
T2’	+lim/-lim	F(3,30)=7.56	P= 0.0004	***	T=-1.38, df=9	P=0.1	**ns**
	dec/dodec	F(3,33)=9.0	P=0.0002	***	T= -4.52, df=10	P=0.0005	***
T3’	+lim/-lim	F(3,70)=76.06	P<0.0001	***	T= -3.55, df=19	P=0.001	***
	dec/dodec	F(3,62)=38.02	P<0.0001	***	T= -6.65, df=17	P<0.0001	***
T4’	+lim/-lim	F(3,13)=3.40	P=0.05	*	T=0.39, df=4	P=0.64	**ns**
	dec/dodec	F(3,27)=10.05	P=0.0001	***	T= -3.71, df=8	P=0.003	**
T5’	+lim/-lim	F(3,18)=21.64	P<0.0001	***	T= -0.37, df=6	P=0.36	**ns**
	dec/dodec	F(3,32)=18.70	P<0.0001	***	T= -3.22, df=10	P=0.004	**

Supplementary Table 2. Statistical results

Group	Odorant	Habituation			Discrimination		
		Repeated measure ANOVA		Significant	T-test (1 tail, paired)		Significant
Group 1	+lim/-lim	F(3,60)=7.35	P=0.0004	***	T = -5.43, df=17	P=0.00002	***
	dec/dodec	F(3,60)=5.8	P=0.0015	**	T= -0.43, df=17	P=0.33	**ns**
Group 2	+lim/-lim	F(3,95)=24.5	p<0.0001	***	T= -1.01, df=27	P=0.15	**ns**
	dec/dodec	F(3,100)=16.73	P<0.0001	***	T= -5.73, df=28	P=0.000002	***
Group 3	+lim/-lim	F(3,67)=13.83	P<0.0001	***	T= -3.01, df=19	P=0.0036	**
	dec/dodec	F(3,67)=12.78	P<0.0001	***	T= -3.34, df=19	P=0.0017	**
Group 4	+lim/-lim	F(3,65)=7.29	P=0.0001	***	T= -2.27, df=19	P=0.018	*
	dec/dodec	F(3,61)=10.42	P<0.0001	***	T= -4.86, df=18	P=0.00006	***

Supplementary Table 3. Statistical results

Group	Odorant	Habituation			Discrimination		
		Repeated measure ANOVA		Significant	T-test (1 tail, paired)	Significant	
Control	+lim/-lim	F(3,53) = 8.65	P= 0.0001	***	T=-1.78, df=15	P=0.048	*
	dec/dodec	F(3,41) = 11.31	p<0.0001	***	T=-3.86, df=12	P=0.001	**
	+lim/+carv	F(3,27) = 9.05	P = 0.0003	***	T=-2.28, df=8	P=0.02	*
Halorhodospin	+lim/-lim	F(3,58) = 8.94	P=0.0001	***	T=-0.12, df=16	P=0.45	**ns**
	dec/dodec	F(3,46) = 6.96	P=0.0006	***	T=-2.24, df=13	P=0.02	*
	+lim/+carv	F(3,46) = 11.42	P<0.0001	***	T=-4.63, df=13	P=0.0002	***

Supplementary Table 4. Statistical results

Supplementary Methods

Mice

Adult C57BL/6J and 129 mice (8 weeks old, male, Charles River, L'arbresles, France) were used in this study. They were housed in standard laboratory cages ('home cage') with water and food ad libitum and were kept on a 12-hr light/dark cycle at a constant temperature of 22°C. All behavioral training was conducted in the afternoon (12:00 – 18:00). Experiments were done following procedures in accordance with the European Community Council Directive of 22nd September 2010 (2010/63/UE) and the National Ethics Committee (Agreement DR2013-48(vM)). Every effort was made to minimize suffering.

Behavior

Experimental Designs

Experiment 1. Mice were injected with a DNA marker, BromodeoxyUridine (BrdU), in order to label a cohort of adult-born neurons. Eight days later, they began the enrichment procedure with (+)limonene (+lim) and (-)limonene (-lim) for one hour daily over 10 days. At the end of the enrichment, mice were tested on their spontaneous discrimination between +lim and -lim and also between another pair of perceptually similar odorants (decanal (dec) and dodecanone (dodec)). Discrimination was assessed using an olfactory habituation/dishabituation task. Animals were sacrificed 24, 34, 44, 54 or 64 days after BrdU injections (Figure 1a).

Experiment 2. Eight days after BrdU injection, a different set of mice were similarly enriched with +lim and -lim but this was followed by a second 10-day enrichment period with dec and dodec either 4, 14, 24, 34 or 44 days after the first. At the end of both enrichments, mice were tested on their spontaneous discrimination between the two odorants of each of the pairs using an olfactory habituation/dishabituation task and animals were sacrificed 24, 34, 44, 54 and 64 days post BrdU injections (Figure 2a).

Experiment 3. We used two other DNA markers (analogues of BrdU), ChlorodeoxyUridine (CldU) and IododeoxyUridine (IdU), to label two different populations of adult-born cells. CldU was injected 8 days before the first enrichment (again with +lim and -lim) while IdU was injected 8 days before the second enrichment period. The second enrichment period varied among groups: no enrichment (group 1); dec and dodec (group 2 and group 4), +lim and -lim plus dec and dodec (group 3). The time between the two enrichments was either 4 or 14 days. As previously, discrimination was assessed using a habituation/dishabituation task and animals were sacrificed 34 or 44 days post-CldU injection. Groups 2 and 4 comprised mice of previous groups T2' and T3' plus 10 additional mice (Figure 3a).

Experiment 4. Using group 3 experimental configuration from the previous experiment, we performed targeted lentiviral-induced halorhodospin channel expression (NpHR3.0) in the subventricular zone, using optogenetics to specifically inhibit the population of adult-born neurons arriving at the beginning of the +lim/-lim enrichment (Figure 4a).

Perceptual learning. This implicit olfactory learning consisted in passive exposure to odorants (enrichment). For the olfactory enrichment, swabs containing 100 µL of pure odorant were placed in two separate tea balls hanging from the cover of the standard home cages for one hour daily over 10 days. For the multiple enrichment (experiment 3, group 3), the two pairs of

odorants were presented with an interval of 1 hour. Odorants used were +lim and -lim as well as dec and dodec (purity >97%, Sigma-Aldrich Corp., St. Louis, MO, USA). The non-enriched control mice were housed and enriched under the same conditions except that the two tea balls contained mineral oil instead of the pure odorants.

Olfactory habituation/dishabituation. We assessed the spontaneous discrimination between two pairs of chemically and perceptually similar odorants: +lim/-lim and dec/dodec. Decanal is an odorant with little overlap with +lim, meaning that the enrichment with one pair of similar odorants should not induce discrimination of the other[1]. The odorants were all diluted in mineral oil proportionally to their vapor pressure in order to reach a pressure of 1 Pa[1]. Experiments were performed in cages similar to the home cage and odorants were presented by placing 60 µL of odor stimulus onto a filter paper (Whatman) which was then placed in a tea ball hanging from the cover of the cage. Each mouse was tested on the two odorant pairs and the odorant pairs were tested in a random order. A test session consisted of one 50-s presentation of mineral oil then four 50-s odor presentations of a first odorant (Hab) at 5 min intervals, followed by one 50-s presentation of the second odorant of the pair (Test). Investigation was defined as active sniffing within 1 cm of the tea ball.

Data analysis. Data analysis was performed using Systat statistical software (SSI, Richmond, CA, USA) and R software (CRAN). Only mice that investigated the tea ball for at least 1 sec during the first presentation of the habituation odorant were included in the analysis (except for the 129 mice in which all mice were included in the analysis due to their low level of exploration). Normality was assessed using the Kolmogorov-Smirnov test. Global two-way ANOVAs were performed to evaluate changes in discrimination abilities between groups. Then intra-group one-way RM-ANOVAs and paired t-tests were performed to determine whether the mice exhibited habituation (trial effect) as well as discrimination (by comparing Hab4 and Test). Discrimination was indicated by a significant increase in investigation time during the test trial. Discrimination index was calculated as [1-(Hab4/Test)]. An index closer to 1 reflects a high ability to discriminate and 0 no discrimination. The criterion for significance was set to $p=0.05$.

Adult-born cells

Proliferative marker administration. To determine the fate of adult-born cells in the OB, Bromodeoxyuridine (BrdU; Sigma-Aldrich B9285), Iododeoxyuridine (IdU; Sigma-Aldrich 17125) and chlorodeoxyuridine (CldU; Sigma-Aldrich C6891) was injected intraperitoneally 8 days before the enrichment period. Three injections of BrdU at 2 h intervals (50 mg/kg in saline) were given[2].

Histology. 24, 34, 44, 54 and 64 days post BrdU injection, five mice were taken randomly from each experimental group and deeply anesthetized by injection of pentobarbital (2 g/kg), and killed with an intracardiac perfusion of 50 mL of cold fixative solution (paraformaldehyde 4% diluted in phosphate-buffered saline, pH 7.4). Brains were then removed, cryoprotected in sucrose (20%) and frozen rapidly before being stored at -20°C. Olfactory bulbs were then sectioned with a cryostat (Reichert-Jung, NuBlock, Germany) into 14µm slices.

BrdU immunohistochemistry. The protocol has been previously described (Moreno et al 2009). Brain sections were first incubated in Target Retrieval Solution (Dako, Trappes, France) for 20 min at 98°C. After cooling for 20 min, they were treated with 0.5% Triton X-100 (Sigma-Aldrich) in PBS for 30 min, then for 3 min with pepsin (0.43 U/ml in 0.1N HCl, Sigma-Aldrich). Endogenous peroxidases were blocked with a solution of 3% H2O2 in 0.1M PBS. Sections were then incubated for 90 min in 5% normal horse serum (Vector Laboratories, Burlingame, CA, USA) in 5% BSA (Sigma-Aldrich) and 0.125% Triton X-100 to block nonspecific binding and then incubated overnight in a mouse anti-BrdU antibody (1:100, Millipore, MAB 4072) at 4°C followed by a biotinylated anti-mouse secondary antibody (1:200, Vector Laboratories, Burlingame, CA, USA) for 2h. The sections were then processed through an avidin-biotin-peroxydase complex (ABC Elite Kit, Vector Laboratories) for 30 min. Finally, sections were reacted in 0.05% 3,3-diaminobenzidine-tetra-hydrochloride (Sigma-Aldrich), 0.03% NiCl2, and 0.03% H2O2 in Tris-HCl buffer (0.05M, pH 7.6), dehydrated in graded ethanols, defatted in xylene and cover-slipped in DPX (Fluka, Sigma-Aldrich).

IdU and CldU immunohistochemistry. Brain sections were incubated in Target Retrieval Solution (Dako, Trappes, France) for 20 min at 98°C, then for 30 min in 0.5% Triton (Sigma-Aldrich) followed with pepsin (0.43 U/ml in 0.1N HCl, Sigma-Aldrich). Sections were then incubated in blocking solution (BSA 2%, Triton 0.1%; horse serum 5% and goat serum 5% in PBS) for 90 min followed with incubation overnight in a rat anti-BrdU/CldU primary antibody (1:100, Millipore, MAB 4072) and a mouse anti-BrdU/IdU primary antibody (1:100, Abcys, ABC 117-7513) in blocking solution at 4°C. Afterwards, sections were incubated for 2h with anti-rat secondary antibody (1:200, Molecular Probes) and anti-mouse secondary antibody (1:200, Vector Laboratories) within a 5% BSA, 5% goat serum, 5% horse serum, PBS solution.

BrdU, IdU and CldU cell assessment. Positive cells were counted on every fifth section (thickness = 14µm, sampling interval = 70µm) in the granule cell layer of the OB using mapping software (Mercator, Explora Nova, La Rochelle, France) coupled to a Zeiss microscope. BrdU-positive cells were manually counted in the granule cell layer. The mean positive cell density of each array was calculated and averaged within each experimental group. Between-groups comparisons of the mean cell density were performed by ANOVA followed by post-hoc t-tests with Tukey corrections. Unilateral t-tests were performed for comparisons between two groups. The level of significance was set to 0.05.

*BrdU/Zif268 experiment.*To investigate immediate early gene expression in response to odorant exposure, mice were presented with a tea ball containing 100µl of pure +lim or dec for 1 hr, 1 hr. before sacrifice. A rabbit anti-Zif268 (1:1000, Santa Cruz Biothechnology) and a rat anti-BrdU (1:100 Harlan Sera-Lab) were used. The appropriate secondary antibodies, coupled to Alexa Fluor 633 and 488 (Invitrogen) were used for revelation of the different markers. Using an Apotome equipped Zeiss fluorescent microscope, at least 30 BrdU-positive cells per animal were examined for co labelling with Zif268. Double labeling was analyzed by pseudoconfocal scanning microscopy using a Zeiss microscope equipped with an Apotome. A percentage of double-labelled cells was calculated for each group and compared using ANOVA followed by t-tests for comparing two groups.

Optogenetics in freely-behaving mice.

Surgery. 150nL of pLenti-hSyn-eNpHR3.0-EYFP lentivirus (9.22 x 10^6 IU/ml) or 300nL of control pLenti-hSyn-EYFP (1.1 x 10^6 IU/ml, expressing only the reporter gene EYFP injections were done bilaterally in the subventricular zone, with the following coordinates respective to bregma: antero-posterior +1mm, medio-lateral ± 1mm, dorso-ventral - 2.3mm and at a rate of 150nL/min. Just after virus infusions, mice were implanted with bilateral optic fibers (200nm core diameter, 0.22 N.A., Doric Lenses) in the olfactory bulb, with the following coordinates respective to bregma: antero-posterior +4.6mm, medio-lateral ±0.75mm, dorso-ventral -2mm. Mice were injected with a ketoprophen solution (2 mg/kg) after the surgery as well as during the following days and allowed to recover with food and water *ad libidum.*

Behavior. During the habituation/dishabituation mice were automatically stimulated (crystal laser, 561nm, 10-15mW, continuous stimulation) only during the test trial (Test) when they entered a 2.5cm diameter zone around the tea ball.

Control of light-triggered inhibition. 36 days post-surgery and lentiviral infusion, mice were stimulated in the hour before sacrifice with light patterns mimicking the average light stimulating pattern during the test trial (0.75s light ON, 5s light OFF for 1h). After brain sectioning (see above), EYFP and Zif268 double immunohistochemistry was performed as described previously: incubation with rabbit Zif268 antibody (1:1.000, Santa Cruz, ref: Sc-189), chicken GFP antibody (1:1.000, Anaspec TEBU, ref: 55423). The density of EYFP, Zif268$^+$ and double-stained cells were counted on 1-2 slices under the injection site to allow assessment of the inhibition. Statistical significance was assessed using a unilateral t-test.

References

1- Moreno, M. M. *et al.* Olfactory perceptual learning requires adult neurogenesis. *Proc Natl Acad Sci U S A* **106**, 17980–17985 (2009).

2- Mandairon, N., Stack, C., Kiselycznyk, C. & Linster, C. Broad activation of the olfactory bulb produces long-lasting changes in odor perception. *Proc Natl Acad Sci U S A* **103**, 13543–13548 (2006)
