# Supplementary material for: Short-term availability of adult-born neurons for memory encoding
Source: Nat Commun. 2019 Dec 6;10:5609. doi: 10.1038/s41467-019-13521-7 (PMC6897887; doi:10.1038/s41467-019-13521-7)
Supplement: Supplementary file 3 — Reporting Summary [file 41467_2019_13521_MOESM3_ESM.pdf]

nature research

Corresponding author(s): *Double-blind peer review submissions: write DBPR and your manuscript number here instead of author names.*

Last updated by author(s): *YYYY-MM-DD*

Reporting Summary

Nature Research wishes to improve the reproducibility of the work that we publish. This form provides structure for consistency and transparency in reporting. For further information on Nature Research policies, see Authors & Referees and the Editorial Policy Checklist.

Statistics

For all statistical analyses, confirm that the following items are present in the figure legend, table legend, main text, or Methods section.

n/a	Confirmed	
☐	☒	The exact sample size (n) for each experimental group/condition, given as a discrete number and unit of measurement
☐	☒	A statement on whether measurements were taken from distinct samples or whether the same sample was measured repeatedly
☐	☒	The statistical test(s) used AND whether they are one- or two-sided<br>*Only common tests should be described solely by name; describe more complex techniques in the Methods section.*
☐	☒	A description of all covariates tested
☐	☒	A description of any assumptions or corrections, such as tests of normality and adjustment for multiple comparisons
☐	☒	A full description of the statistical parameters including central tendency (e.g. means) or other basic estimates (e.g. regression coefficient) AND variation (e.g. standard deviation) or associated estimates of uncertainty (e.g. confidence intervals)
☐	☒	For null hypothesis testing, the test statistic (e.g. F, t, r) with confidence intervals, effect sizes, degrees of freedom and P value noted<br>*Give P values as exact values whenever suitable.*
☒	☐	For Bayesian analysis, information on the choice of priors and Markov chain Monte Carlo settings
☒	☐	For hierarchical and complex designs, identification of the appropriate level for tests and full reporting of outcomes
☐	☒	Estimates of effect sizes (e.g. Cohen's d, Pearson's r), indicating how they were calculated

Our web collection on statistics for biologists contains articles on many of the points above.

Software and code

Policy information about availability of computer code

Data collection	Codes are provided in a supplementary file
Data analysis	We used R software (CRAN)

For manuscripts utilizing custom algorithms or software that are central to the research but not yet described in published literature, software must be made available to editors/reviewers. We strongly encourage code deposition in a community repository (e.g. GitHub). See the Nature Research guidelines for submitting code & software for further information.

Data

Policy information about availability of data

All manuscripts must include a data availability statement. This statement should provide the following information, where applicable:

- Accession codes, unique identifiers, or web links for publicly available datasets
- A list of figures that have associated raw data
- A description of any restrictions on data availability

The data associated to the figures and the codes used are available as supplementary files.

Field-specific reporting

Please select the one below that is the best fit for your research. If you are not sure, read the appropriate sections before making your selection.

☒ Life sciences ☐ Behavioural & social sciences ☐ Ecological, evolutionary & environmental sciences

For a reference copy of the document with all sections, see nature.com/documents/nr-reporting-summary-flat.pdf

Life sciences study design

All studies must disclose on these points even when the disclosure is negative.

Sample size	No statistical methods were used to predetermine sample sizes, but our sample sizes were similar to those reported in previous publications (Moreno et al 2009 PNAS; Forest et al 2019 Cerebral Cortex, Mandairon et al 2018 eLife)
Data exclusions	Regarding habituation.dishabituation test, only mice that investigated the tea ball for at least 1 sec during the first presentation of the habituation odorant were included in the analysis, except in the 129 mice experiment where no data were excluded.
Replication	The habituation/dishabituation task in controls replicates previous studies. The same is true for cells counts. In addition, procedures and criteria for behavioral evaluation and labelling or co-labelling counts are established in the lab and used systematically, regardless of the experiment or experimental group.
Randomization	Randomization was used to form experimental groups. No particular method was used.
Blinding	All cell counts were conducted blind with regard to the experimental group. Mice exploration assessments were done blind with regard to the experimental group.

Reporting for specific materials, systems and methods

We require information from authors about some types of materials, experimental systems and methods used in many studies. Here, indicate whether each material, system or method listed is relevant to your study. If you are not sure if a list item applies to your research, read the appropriate section before selecting a response.

Materials & experimental systems

n/a	Involved in the study	
☐	☒	Antibodies
☒	☐	Eukaryotic cell lines
☒	☐	Palaeontology
☐	☒	Animals and other organisms
☒	☐	Human research participants
☒	☐	Clinical data

Methods

n/a	Involved in the study	
☒	☐	ChIP-seq
☒	☐	Flow cytometry
☒	☐	MRI-based neuroimaging

Antibodies

Antibodies used	rabbit Zif268 antibody (1:1.000, Santa Cruz, ref: Sc-189), chicken GFP antibody (1:1.000, Anaspec TEBU, ref: 55423), mouse anti-BrdU antibody (1:100, Millipore, MAB 4072) , rat anti-CldU primary antibody (1:100, Millipore, MAB 4072), mouse anti-Idu primary antibody (1:100, Abcys, ABC 117-7513)
Validation	Validation Zif268 and GFP: see Kermen et al 2016 Nature Neuroscience<br>Validation BrdU see Forest et al Cerebral Cortex 2019 and Mandairon et al eLife 2018

Animals and other organisms

Policy information about studies involving animals; ARRIVE guidelines recommended for reporting animal research

Laboratory animals	Adult C57BL/6J mice (8 weeks old, male, Charles River, L'arbresles, France) were used in this study.
Wild animals	NA
Field-collected samples	NA
Ethics oversight	Experiments were done following procedures in accordance with the European Community Council Directive of 22nd September 2010 (2010/63/UE) and approved by the National Ethics Committee (Agreement DR2013-48(vM)).

Note that full information on the approval of the study protocol must also be provided in the manuscript.
