# Supplementary material for: Short-term availability of adult-born neurons for memory encoding
Source: Nat Commun. 2019 Dec 6;10:5609. doi: 10.1038/s41467-019-13521-7 (PMC6897887; doi:10.1038/s41467-019-13521-7)
Supplement: Supplementary file 4 — Description of Additional Supplementary Files [file 41467_2019_13521_MOESM4_ESM.pdf]

Description of Additional Supplementary Files

File Name: Supplementary Software 1
Description: Codes used in the manuscript.
