# Supplementary material for: Short-term availability of adult-born neurons for memory encoding
Source: Nat Commun. 2019 Dec 6;10:5609. doi: 10.1038/s41467-019-13521-7 (PMC6897887; doi:10.1038/s41467-019-13521-7)
Supplement: Supplementary file 5 — Supplementary Software 1 [file 41467_2019_13521_MOESM5_ESM.pdf]

```
rm(list = ls())

##### Library ######
library(openxlsx)
library(ggplot2)
library(plyr)
library(reshape2)
library(nlme)
library(multcomp)
library(lsmeans)

setwd("/media/jeremy/Data/CloudStation/temporaire/papier changeant/data_csv")


######    MANIP 1    ######
######    Reading datasets ######

dataset.lim.T1NE <- read.csv("dataset_lim_T1NE.csv", header=T, sep = ",")
dataset.lim.T1 <- read.csv("dataset_lim_T1.csv", header=T, sep = ",")
dataset.lim.T2 <- read.csv("dataset_lim_T2.csv", header=T, sep = ",")
dataset.lim.T3 <- read.csv("dataset_lim_T3.csv", header=T, sep = ",")
dataset.lim.T4 <- read.csv("dataset_lim_T4.csv", header=T, sep = ",")
dataset.lim.T5 <- read.csv("dataset_lim_T5.csv", header=T, sep = ",")

dataset.dd.T1NE <- read.csv("dataset_dd_T1NE.csv", header=T, sep = ",")
dataset.dd.T1 <- read.csv("dataset_dd_T1.csv", header=T, sep = ",")
dataset.dd.T2 <- read.csv("dataset_dd_T2.csv", header=T, sep = ",")
dataset.dd.T3 <- read.csv("dataset_dd_T3.csv", header=T, sep = ",")
dataset.dd.T4 <- read.csv("dataset_dd_T4.csv", header=T, sep = ",")
dataset.dd.T5 <- read.csv("dataset_dd_T5.csv", header=T, sep = ",")


dataset.lim <- rbind.fill(dataset.lim.T1NE, dataset.lim.T1, dataset.lim.T2, dataset.lim.T3,
dataset.lim.T4, dataset.lim.T5)
dataset.lim <- na.omit(dataset.lim)
dataset.lim <- dataset.lim[, c("Hab1","Hab2", "Hab3", "Hab4", "Test", "groupe", "X")]
dataset.lim.discri <- dataset.lim
dataset.lim <- melt(dataset.lim,
measure.vars=c("Hab1","Hab2","Hab3","Hab4","Test"),factorAsStrings=F)
names(dataset.lim)[2]<-"souris"
names(dataset.lim)[3]<-"session"
names(dataset.lim)[4]<-"temps"


dataset.dd <- rbind.fill(dataset.dd.T1NE, dataset.dd.T1, dataset.dd.T2, dataset.dd.T3, dataset.dd.T4,
dataset.dd.T5)
dataset.dd <- na.omit(dataset.dd)
dataset.dd <- dataset.dd[, c("Hab1","Hab2", "Hab3", "Hab4", "Test", "groupe", "X")]

dataset.dd <- melt(dataset.dd,
measure.vars=c("Hab1","Hab2","Hab3","Hab4","Test"),factorAsStrings=F)
names(dataset.dd)[2]<-"souris"
```

```
names(dataset.dd)[3]<-"session"
names(dataset.dd)[4]<-"temps"

##########################################################################################
##########################
############################################     statistiques
############################################
##########################################################################################
##########################

######### BEHAVIOR ########
##### LIM
GROUPE <- c("lim_T1NE", "lim_T1", "lim_T2", "lim_T3", "lim_T4", "lim_T5")

for (groupe in GROUPE){
  print(paste0("Resultats pour ", groupe))
  dataset.lim.groupe.hab <- dataset.lim[(dataset.lim$groupe==groupe &
(dataset.lim$session=="Hab1"|
                                        dataset.lim$session=="Hab2"|
                                        dataset.lim$session=="Hab3"|
                                        dataset.lim$session=="Hab4")),]
  dataset.lim.groupe.hab <- droplevels(dataset.lim.groupe.hab)
  dataset.lim.groupe.hab.lme <- lme(temps~session,random=~1|souris,
data=dataset.lim.groupe.hab);
  print(anova(dataset.lim.groupe.hab.lme))

  dataset.lim.groupe.discri <- dataset.lim[(dataset.lim$groupe==groupe &
(dataset.lim$session=="Hab4"|
                                           dataset.lim$session=="Test")),]
  #var.test(dataset.lim.groupe.discri[dataset.lim.groupe.discri$session=='Hab4',]$temps,
  #       dataset.lim.groupe.discri[dataset.lim.groupe.discri$session=='Test',]$temps)
  print(t.test(dataset.lim.groupe.discri[dataset.lim.groupe.discri$session=='Hab4',]$temps,
             dataset.lim.groupe.discri[dataset.lim.groupe.discri$session=='Test',]$temps,
             alternative = c("less"), var.equal = TRUE, paired = TRUE))
  readline(prompt="Press enter to continue")
}


##### DD
GROUPE2 <- c("dd_T1NE", "dd_T1", "dd_T2", "dd_T3", "dd_T4", "dd_T5")

for (groupe in GROUPE2){
  print(paste0("Resultats pour ", groupe))
  dataset.dd.groupe.hab <- dataset.dd[(dataset.dd$groupe==groupe & (dataset.dd$session=="Hab1"|
                                       dataset.dd$session=="Hab2"|
                                       dataset.dd$session=="Hab3"|
                                       dataset.dd$session=="Hab4")),]
  dataset.dd.groupe.hab <- droplevels(dataset.dd.groupe.hab)
  dataset.dd.groupe.hab.lme <- lme(temps~session,random=~1|souris, data=dataset.dd.groupe.hab);
  print(anova(dataset.dd.groupe.hab.lme))
```

```
  dataset.dd.groupe.discri <- dataset.dd[(dataset.dd$groupe==groupe &
(dataset.dd$session=="Hab4"|
                                                             dataset.dd$session=="Test")),]
  #var.test(dataset.dd.groupe.discri[dataset.dd.groupe.discri$session=='Hab4',]$temps,
  #         dataset.dd.groupe.discri[dataset.dd.groupe.discri$session=='Test',]$temps)
  print(t.test(dataset.dd.groupe.discri[dataset.dd.groupe.discri$session=='Hab4',]$temps,
             dataset.dd.groupe.discri[dataset.dd.groupe.discri$session=='Test',]$temps,
             alternative = c("less"), var.equal = TRUE, paired = TRUE))
  readline(prompt="Press enter to continue")
}

########################################################################################
#########################
###########################################     graphiques
###########################################
########################################################################################
#########################

### LIMONENE
infodata.dataset.lim<-ddply(dataset.lim,c("groupe", "session"),summarise,
                        N = length(temps), mean=mean(temps), sd=sd(temps), sem=sd/sqrt(N))

graph.dataset.lim.manip1<-ggplot((data=infodata.dataset.lim[(infodata.dataset.lim$session ==
"Hab4" | infodata.dataset.lim$session == "Test"),]),
                            aes(x=groupe,y=mean, fill=session)) +
  geom_jitter(data = dataset.lim[(dataset.lim$session == "Hab4" | dataset.lim$session == "Test"),],
             aes(x=groupe,y=temps),size=1.5,
             show.legend = FALSE, position = position_jitterdodge(jitter.width = 0.25, jitter.height = 0,
dodge.width = 0.6)) +
  geom_bar(stat="identity", show.legend=TRUE, position=position_dodge(width=0.6), colour =
"black", width=0.6, alpha=0.7)+
  geom_errorbar(aes(ymin=mean,ymax=mean+sem, width=0.3),
position=position_dodge(width=0.6))+
  scale_x_discrete(labels = c("T1NE", "T1", "T2", "T3", "T4", "T5"))+
  scale_fill_manual(values=c("grey80", "grey30"), label = c("Hab4", 'Test'))+
  scale_y_continuous("Investigation time (s)",
                   limits=c(-0.001,6), breaks=c(0,1,2,3,4,5,6),
                   expand = c(0,0))+
  labs(title="(+)limonene / (-)limonene")+
  theme(panel.background=element_rect(fill="white"),plot.title=element_text(size=18),
        axis.line.x=element_line(color = "black", size= .5), axis.line.y = element_line(color = "black",
size = .5), axis.ticks=element_line(size=2),
        axis.title.y=element_text(size=22), axis.title.x=element_text(size=0),
        axis.text.x=element_text(size=18, colour="black"), axis.text.y=element_text(size=18,
colour="black"),
        panel.grid.minor=element_blank(), panel.grid.major=element_blank(),
        legend.title=element_blank())
print(graph.dataset.lim.manip1)
ggsave("D:/Jerem/Documents/Manip Lyon/perceptif changeant/graph_dataset_lim_manip1.svg")

### DEC DODEC
infodata.dataset.dd<-ddply(dataset.dd,c("groupe", "session"),summarise,
```

```
                    N = length(temps), mean=mean(temps), sd=sd(temps), sem=sd/sqrt(N))

graph.dataset.dd.manip1<-ggplot((data=infodata.dataset.dd[(infodata.dataset.dd$session == "Hab4"
| infodata.dataset.dd$session == "Test"),]),
                        aes(x=groupe,y=mean, fill=session)) +
  geom_jitter(data = dataset.dd[(dataset.dd$session == "Hab4" | dataset.dd$session == "Test"),],
            aes(x=groupe,y=temps),size=1.5,
            show.legend = FALSE, position = position_jitterdodge(jitter.width = 0.25, jitter.height = 0,
dodge.width = 0.6)) +
  geom_bar(stat="identity", show.legend=TRUE, position=position_dodge(width=0.6), colour =
"black", width=0.6, alpha=0.7)+
  geom_errorbar(aes(ymin=mean,ymax=mean+sem, width=0.3),
position=position_dodge(width=0.6))+
  scale_x_discrete(labels = c("T1NE", "T1", "T2", "T3", "T4", "T5"))+
  scale_fill_manual(values=c("grey80", "grey30"), label = c("Hab4", 'Test'))+
  scale_y_continuous("Investigation time (s)",
                limits=c(-0.001,6), breaks=c(0,1,2,3,4,5,6),
                expand = c(0,0))+
  labs(title="decanal / dodecanone")+
  theme(panel.background=element_rect(fill="white"),plot.title=element_text(size=18),
        axis.line.x=element_line(color = "black", size= .5), axis.line.y = element_line(color = "black",
size = .5), axis.ticks=element_line(size=2),
        axis.title.y=element_text(size=22), axis.title.x=element_text(size=0),
        axis.text.x=element_text(size=18, colour="black"), axis.text.y=element_text(size=18,
colour="black"),
        panel.grid.minor=element_blank(), panel.grid.major=element_blank(),
        legend.title=element_blank())
print(graph.dataset.dd.manip1)
ggsave("D:/Jerem/Documents/Manip Lyon/perceptif changeant/graph_dataset_dd_manip1.svg")

################################################################################
#########################
##########################################     graphiques habituation
##########################################
################################################################################
#########################

### LIMONENE
graph.dataset.lim.manip1.habituation<-
ggplot((data=infodata.dataset.lim[(infodata.dataset.lim$session == "Hab1" |
                                                   infodata.dataset.lim$session == "Hab2" |
                                                   infodata.dataset.lim$session == "Hab3" |
                                                   infodata.dataset.lim$session == "Hab4"),]),
                              aes(x=groupe,y=mean, fill=session)) +
  geom_jitter(data = dataset.lim[(dataset.lim$session == "Hab1" |
                          dataset.lim$session == "Hab2" |
                          dataset.lim$session == "Hab3" |
                          dataset.lim$session == "Hab4"),],
            aes(x=groupe,y=temps),size=1.5,
            show.legend = FALSE, position = position_jitterdodge(jitter.width = 0.25, jitter.height = 0,
dodge.width = 0.6)) +
```

```
  geom_bar(stat="identity", show.legend=TRUE, position=position_dodge(width=0.6), colour =
"black", width=0.6, alpha=0.7)+
  geom_errorbar(aes(ymin=mean,ymax=mean+sem, width=0.3),
position=position_dodge(width=0.6))+
  scale_x_discrete(labels = c("T1NE", "T1", "T2", "T3", "T4", "T5"))+
  scale_fill_manual(values=c("grey50", "grey60", "grey70", "grey80"), label =
c("Hab1","Hab2","Hab3","Hab4"))+
  scale_y_continuous("Investigation time (s)",
                     limits=c(-0.001,9), breaks=c(0,1,2,3,4,5,6,7,8,9),
                     expand = c(0,0))+
  labs(title="(+)limonene / (-)limonene")+
  theme(panel.background=element_rect(fill="white"),plot.title=element_text(size=18),
        axis.line.x=element_line(color = "black", size= .5), axis.line.y = element_line(color = "black",
size = .5), axis.ticks=element_line(size=2),
        axis.title.y=element_text(size=22), axis.title.x=element_text(size=0),
        axis.text.x=element_text(size=18, colour="black"), axis.text.y=element_text(size=18,
colour="black"),
        panel.grid.minor=element_blank(), panel.grid.major=element_blank(),
        legend.title=element_blank())
print(graph.dataset.lim.manip1.habituation)
ggsave("D:/Jerem/Documents/Manip Lyon/perceptif
changeant/graph_dataset_lim_manip1.habituation.svg")

### DEC DODEC

graph.dataset.dd.manip1.habituation<-ggplot((data=infodata.dataset.dd[(infodata.dataset.dd$session
== "Hab1" |
                                                                        infodata.dataset.dd$session == "Hab2" |
                                                                        infodata.dataset.dd$session == "Hab3" |
                                                                        infodata.dataset.dd$session == "Hab4"),]),
                                            aes(x=groupe,y=mean, fill=session)) +
  geom_jitter(data = dataset.dd[(dataset.dd$session == "Hab1" |
                                 dataset.dd$session == "Hab2" |
                                 dataset.dd$session == "Hab3" |
                                 dataset.dd$session == "Hab4"),],
              aes(x=groupe,y=temps),size=1.5,
              show.legend = FALSE, position = position_jitterdodge(jitter.width = 0.25, jitter.height = 0,
dodge.width = 0.6)) +
  geom_bar(stat="identity", show.legend=TRUE, position=position_dodge(width=0.6), colour =
"black", width=0.6, alpha=0.7)+
  geom_errorbar(aes(ymin=mean,ymax=mean+sem, width=0.3),
position=position_dodge(width=0.6))+
  scale_x_discrete(labels = c("T1NE", "T1", "T2", "T3", "T4", "T5"))+
  scale_fill_manual(values=c("grey50", "grey60", "grey70", "grey80"), label =
c("Hab1","Hab2","Hab3","Hab4"))+
  scale_y_continuous("Investigation time (s)",
                     limits=c(-0.001,9), breaks=c(0,1,2,3,4,5,6,7,8,9),
                     expand = c(0,0))+
  labs(title="decanal / dodecanone")+
  theme(panel.background=element_rect(fill="white"),plot.title=element_text(size=18),
        axis.line.x=element_line(color = "black", size= .5), axis.line.y = element_line(color = "black",
size = .5), axis.ticks=element_line(size=2),
```

```
        axis.title.y=element_text(size=22), axis.title.x=element_text(size=0),
        axis.text.x=element_text(size=18, colour="black"), axis.text.y=element_text(size=18,
colour="black"),
        panel.grid.minor=element_blank(), panel.grid.major=element_blank(),
        legend.title=element_blank())
print(graph.dataset.dd.manip1.habituation)
ggsave("D:/Jerem/Documents/Manip Lyon/perceptif
changeant/graph_dataset_dd_manip1.habituation.svg")
ggsave("/media/jeremy/Data/CloudStation/temporaire/papier
changeant/new_data_for_review/graph_dataset_dd_manip1.habituation.svg",
       width = 90, height=90, units = c("mm"))

########    BRDU     ########
dataset.brdu.manip1 <- read.csv("brdu.csv", header=T, sep = ",", dec=',')

dataset.brdu.manip1.lm <- lm(densite~group, data=dataset.brdu.manip1)
print(anova(dataset.brdu.manip1.lm))

## regression sur les groupes actifs
dataset.brdu.manip1.noNE <- dataset.brdu.manip1[(dataset.brdu.manip1$group == 'T1' |
dataset.brdu.manip1$group == 'T2' |
                                                  dataset.brdu.manip1$group == 'T3' | dataset.brdu.manip1$group ==
'T4' |
                                                  dataset.brdu.manip1$group == 'T5'),]
dataset.brdu.manip1.noNE.lm <- lm(densite~group, data=dataset.brdu.manip1.noNE)
print(summary(dataset.brdu.manip1.noNE.lm))
print(anova(dataset.brdu.manip1.noNE.lm))

print(pairwise.t.test(dataset.brdu.manip1$densite, dataset.brdu.manip1$group, p.adjust.method =
"holm", pool.sd = FALSE, alternative = "less"))

# Separation discri vs non discri vs control au niveau comportement
dataset.brdu.manip1$discri <- c("control","control",
                                "oui","oui","oui","oui","oui","oui","oui","oui","oui","oui","oui","oui",
                                "non","non","non","non","non","non","non","non")
dataset.brdu.manip1$discri <- as.factor(dataset.brdu.manip1$discri)

t.test(dataset.brdu.manip1[dataset.brdu.manip1$discri=='oui',]$densite,
dataset.brdu.manip1[dataset.brdu.manip1$discri=='non',]$densite)

## Correlation index discri et brdu densite
dataset.lim.discri$index <- 1-(dataset.lim.discri$Hab4 / dataset.lim.discri$Test)  ## reprendre
dataset en large (en haut)
for (i in seq(dataset.lim.discri$index)){
  if (is.nan(dataset.lim.discri$index[i])){
    dataset.lim.discri$index[i] <- 0
  }
  else if(dataset.lim.discri$index[i] == -Inf){
    dataset.lim.discri$index[i] <- 0
  }
  else if (dataset.lim.discri$index[i] < 0){
    dataset.lim.discri$index[i] <- 0
```

```
  }
  else {
    dataset.lim.discri$index[i] <- dataset.lim.discri$index[i]
  }
}

dataset.lim.discri.summary<-ddply(dataset.lim.discri,c("groupe"),summarise,
                           N = length(index), mean=mean(index), sd=sd(index), sem=sd/sqrt(N))
dataset.brdu.manip1.summary <- ddply(dataset.brdu.manip1,c("group"),summarise,
                            N = length(densite), mean=mean(densite), sd=sd(densite), sem=sd/sqrt(N))
dataset.correlation <- data.frame(index =
c((dataset.lim.discri.summary[dataset.lim.discri.summary$groupe == 'lim_T1NE',]$mean),
                                  (dataset.lim.discri.summary[dataset.lim.discri.summary$groupe ==
'lim_T1',]$mean),
                                  (dataset.lim.discri.summary[dataset.lim.discri.summary$groupe ==
'lim_T2',]$mean),
                                  (dataset.lim.discri.summary[dataset.lim.discri.summary$groupe ==
'lim_T3',]$mean),
                                  (dataset.lim.discri.summary[dataset.lim.discri.summary$groupe ==
'lim_T4',]$mean),
                                  (dataset.lim.discri.summary[dataset.lim.discri.summary$groupe ==
'lim_T5',]$mean)),
                           densite =
c((dataset.brdu.manip1.summary[dataset.brdu.manip1.summary$group == 'T1NE',]$mean),
                                   (dataset.brdu.manip1.summary[dataset.brdu.manip1.summary$group
== 'T1',]$mean),
                                   (dataset.brdu.manip1.summary[dataset.brdu.manip1.summary$group
== 'T2',]$mean),
                                   (dataset.brdu.manip1.summary[dataset.brdu.manip1.summary$group
== 'T3',]$mean),
                                   (dataset.brdu.manip1.summary[dataset.brdu.manip1.summary$group
== 'T4',]$mean),
                                   (dataset.brdu.manip1.summary[dataset.brdu.manip1.summary$group
== 'T5',]$mean)),
                           index_sem =
c((dataset.lim.discri.summary[dataset.lim.discri.summary$groupe == 'lim_T1NE',]$sem),
                                    (dataset.lim.discri.summary[dataset.lim.discri.summary$groupe ==
'lim_T1',]$sem),
                                    (dataset.lim.discri.summary[dataset.lim.discri.summary$groupe ==
'lim_T2',]$sem),
                                    (dataset.lim.discri.summary[dataset.lim.discri.summary$groupe ==
'lim_T3',]$sem),
                                    (dataset.lim.discri.summary[dataset.lim.discri.summary$groupe ==
'lim_T4',]$sem),
                                    (dataset.lim.discri.summary[dataset.lim.discri.summary$groupe ==
'lim_T5',]$sem)),
                           densite_sem =
c((dataset.brdu.manip1.summary[dataset.brdu.manip1.summary$group == 'T1NE',]$sem),

(dataset.brdu.manip1.summary[dataset.brdu.manip1.summary$group == 'T1',]$sem),

(dataset.brdu.manip1.summary[dataset.brdu.manip1.summary$group == 'T2',]$sem),
```

```
(dataset.brdu.manip1.summary[dataset.brdu.manip1.summary$group == 'T3',]$sem),

(dataset.brdu.manip1.summary[dataset.brdu.manip1.summary$group == 'T4',]$sem),

(dataset.brdu.manip1.summary[dataset.brdu.manip1.summary$group == 'T5',]$sem)))

print(cor.test(dataset.correlation$index, dataset.correlation$densite))

## GRAPH BRDU
## GROUPED ACCORDING TO DISCRI OR NO DISCRI
infodata.brdu.manip1.grouped <- ddply(dataset.brdu.manip1,c("discri"),summarise,
                                      N = length(densite), mean=mean(densite), sd=sd(densite), sem=sd/sqrt(N))

infodata.brdu.manip1.grouped$mean <- infodata.brdu.manip1.grouped$mean*100000
infodata.brdu.manip1.grouped$sem <- infodata.brdu.manip1.grouped$sem*100000

dataset.brdu.manip1$densite <- dataset.brdu.manip1$densite*100000

graph.dataset.brdu.grouped.manip1<-
ggplot((data=infodata.brdu.manip1.grouped[(infodata.brdu.manip1.grouped$discri == "control" |
                                                                    infodata.brdu.manip1.grouped$discri == "non")|
                                                                   infodata.brdu.manip1.grouped$discri == "oui",]),
                                          aes(x=discri,y=mean, fill=discri)) +
  geom_jitter(data = dataset.brdu.manip1[(dataset.brdu.manip1$discri == "control" |
dataset.brdu.manip1$discri == "non" |
                                            dataset.brdu.manip1$discri == "oui" ),],
              aes(x=discri,y=densite),size=1.5, show.legend = FALSE,
              position = position_jitterdodge(jitter.width = 0.25, jitter.height = 0, dodge.width = 0.6)) +
  geom_bar(stat="identity", show.legend=TRUE, position=position_dodge(width=0.6), colour =
"black", width=0.6, alpha=0.7)+
  geom_errorbar(aes(ymin=mean,ymax=mean+sem, width=0.3),
position=position_dodge(width=0.6))+
  scale_x_discrete(labels = c("T1NE", "No discrimation", "Disrimination"))+
  scale_fill_manual(values=c("grey90", "grey70", "grey30"), label = c("Control", 'Non-Enriched',
'Enriched'))+
  scale_y_continuous("Brdu-positive cell density (cell/?m? *" ~ 10^-05 ~")",
                     limits=c(-0.0001,7.5), breaks=c(0,1,2,3,4,5,6,7),
                     expand = c(0,0))+
  labs(title="")+
  theme(panel.background=element_rect(fill="white"),plot.title=element_text(size=18),
        axis.line.x=element_line(color = "black", size= .5), axis.line.y = element_line(color = "black",
size = .5), axis.ticks=element_line(size=2),
        axis.title.y=element_text(size=22), axis.title.x=element_text(size=0),
        axis.text.x=element_text(angle=60, hjust=1,size=18, colour="black"),
axis.text.y=element_text(size=18, colour="black"),
        panel.grid.minor=element_blank(), panel.grid.major=element_blank(),
        legend.title=element_blank())+
  annotate(geom = 'text', label = '*', x = 3, y = 7, size = 15)
print(graph.dataset.brdu.grouped.manip1)
#ggsave("D:/Jerem/Documents/Manip Lyon/perceptif
changeant/graph_dataset_brdu_grouped_manip1.svg", width=7, height=11)
```

```
ggsave("N:/neuropop/J?r?my/J?r?my/Recherches/Th?se + Stage M2/Recherches/Manip
apprentissage perceptif changeant/graph_dataset_brdu_grouped_manip1.svg", width=7, height=11)

### GROUPED ACCORDING TO GROUPS
infodata.brdu.manip1 <- ddply(dataset.brdu.manip1,c("group"),summarise,
                              N = length(densite), mean=mean(densite), sd=sd(densite), sem=sd/sqrt(N))

infodata.brdu.manip1$group <- factor(infodata.brdu.manip1$group, levels=c("T1NE", "T1", "T2",
"T3", "T4", "T5"))
dataset.brdu.manip1$group <- factor(dataset.brdu.manip1$group, levels=c("T1NE", "T1", "T2",
"T3", "T4", "T5"))

graph.dataset.brdu.manip1<-ggplot((data=infodata.brdu.manip1),aes(x=group,y=mean, fill=group))
+
  geom_jitter(data = dataset.brdu.manip1,
              aes(x=group,y=densite),size=1.5, show.legend = FALSE,
              position = position_jitterdodge(jitter.width = 0.25, jitter.height = 0, dodge.width = 0.6)) +
  geom_bar(stat="identity", show.legend=FALSE, position=position_dodge(width=0.6), colour =
"black", width=0.6, alpha=0.7)+
  geom_errorbar(aes(ymin=mean,ymax=mean+sem, width=0.3),
position=position_dodge(width=0.6))+
  scale_x_discrete(labels = c("T1NE", "T1", "T2", "T3", "T4", "T5"))+
  scale_fill_manual(values=c("grey90", "grey30", "grey30", "grey30", "grey30", "grey30"))+
  scale_y_continuous("Brdu-positive cell density (cell *" ~ 10^-05 ~" /?m?)",
                     limits=c(-0.0001,70), breaks=c(0,10,20,30,40,50,60,70),
                     expand = c(0,0))+
  labs(title="")+
  theme(panel.background=element_rect(fill="white"),plot.title=element_text(size=18),
        axis.line.x=element_line(color = "black", size= .5), axis.line.y = element_line(color = "black",
size = .5), axis.ticks=element_line(size=2),
        axis.title.y=element_text(size=22), axis.title.x=element_text(size=0),
        axis.text.x=element_text(angle=0, hjust=0.5,size=18, colour="black"),
axis.text.y=element_text(size=18, colour="black"),
        panel.grid.minor=element_blank(), panel.grid.major=element_blank(),
        legend.title=element_blank())
print(graph.dataset.brdu.manip1)
ggsave("D:/Jerem/Documents/Manip Lyon/perceptif changeant/graph_dataset_brdu_manip1..pdf")
ggsave("N:/neuropop/J?r?my/J?r?my/Recherches/Th?se + Stage M2/Recherches/Manip
apprentissage perceptif changeant/graph_dataset_brdu_manip1.svg", width=7, height=11)

#### GRAPH CORRELATION
dataset.correlation$densite <- dataset.correlation$densite*100000
dataset.correlation$densite_sem <- dataset.correlation$densite_sem*100000


graph.correlation.manip1<- ggplot(data=dataset.correlation, aes(x = index, y = densite))+
  geom_point(size = 3, colour = "black")+
  geom_errorbar(data = dataset.correlation, aes(ymin=densite-densite_sem, ymax =
densite+densite_sem, width=0.001))+
  geom_errorbarh(data = dataset.correlation, aes(xmin=index-index_sem, xmax = index+index_sem,
height = 0.001))+
  geom_smooth(colour="black", method="lm", size=1, se=F)+
```

```
  scale_x_continuous("Discrimination index")+
  scale_y_continuous("Brdu-positive cell density (cell/?m? *" ~ 10^-05 ~")",
                     limits=c(2.5,6), breaks=c(3,4,5,6),
                     expand = c(0,0))+
  labs(title="")+
  theme(panel.background=element_rect(fill="white"),plot.title=element_text(size=18),
        axis.line.x=element_line(color = "black", size= .5), axis.line.y = element_line(color = "black",
size = .5), axis.ticks=element_line(size=2),
        axis.title.y=element_text(size=26), axis.title.x=element_text(size=26),
        axis.text.x=element_text(angle=0, hjust=0.5,size=22, colour="black"),
axis.text.y=element_text(size=22, colour="black"),
        panel.grid.minor=element_blank(), panel.grid.major=element_blank(),
        legend.title=element_blank())



print(graph.correlation.manip1)
ggsave("D:/Jerem/Documents/Manip Lyon/perceptif changeant/graph_correlation_manip1.pdf")
ggsave("N:/neuropop/J?r?my/J?r?my/Recherches/Th?se + Stage M2/Recherches/Manip
apprentissage perceptif changeant/graph_correlation_manip1.pdf", width=7, height=11)

##### MANIP 2

######   Reading datasets ######

dataset2.lim.T2prime <- read.csv("dataset_lim_T2_prime.csv", header=T, sep = ",")
dataset2.lim.T3prime <- read.csv("dataset_lim_T3_prime.csv", header=T, sep = ",")
dataset2.lim.T4prime <- read.csv("dataset_lim_T4_prime.csv", header=T, sep = ",")
dataset2.lim.T5prime <- read.csv("dataset_lim_T5_prime.csv", header=T, sep = ",")

dataset2.dd.T2prime <- read.csv("dataset_dd_T2_prime.csv", header=T, sep = ",")
dataset2.dd.T3prime <- read.csv("dataset_dd_T3_prime.csv", header=T, sep = ",")
dataset2.dd.T4prime <- read.csv("dataset_dd_T4_prime.csv", header=T, sep = ",")
dataset2.dd.T5prime <- read.csv("dataset_dd_T5_prime.csv", header=T, sep = ",")

dataset2.lim <- rbind.fill(dataset.lim.T1, dataset2.lim.T2prime, dataset2.lim.T3prime,
dataset2.lim.T3prime,
                           dataset2.lim.T4prime, dataset2.lim.T5prime)
dataset2.lim <- na.omit(dataset2.lim)
dataset2.lim <- dataset2.lim[, c("Hab1","Hab2", "Hab3", "Hab4", "Test", "groupe", "X")]
dataset2.lim.discri <- dataset2.lim
dataset2.lim <- melt(dataset2.lim,
measure.vars=c("Hab1","Hab2","Hab3","Hab4","Test"),factorAsStrings=F)
names(dataset2.lim)[2]<-"souris"
names(dataset2.lim)[3]<-"session"
names(dataset2.lim)[4]<-"temps"

dataset2.dd <- rbind.fill(dataset.dd.T1, dataset2.dd.T2prime, dataset2.dd.T3prime,
dataset2.dd.T3prime,
                          dataset2.dd.T4prime, dataset2.dd.T5prime)
dataset2.dd <- na.omit(dataset2.dd)
dataset2.dd <- dataset2.dd[, c("Hab1","Hab2", "Hab3", "Hab4", "Test", "groupe", "X")]
```

```
dataset2.dd <- melt(dataset2.dd,
measure.vars=c("Hab1","Hab2","Hab3","Hab4","Test"),factorAsStrings=F)
names(dataset2.dd)[2]<-"souris"
names(dataset2.dd)[3]<-"session"
names(dataset2.dd)[4]<-"temps"

###############################################################################
########################
########################################    statistiques
########################################
###############################################################################
########################

dataset2.lim.discri <- dataset2.lim[(dataset2.lim$session == 'Hab4'|dataset2.lim$session == 'Test'),]
dataset2.lim.discri.lm <- lm(temps~session*groupe, data=dataset2.lim.discri)
print(anova(dataset2.lim.discri.lm))


dataset2.dd.discri <- dataset2.dd[(dataset2.dd$session == 'Hab4'|dataset2.dd$session == 'Test'),]
dataset2.dd.discri.lm <- lm(temps~groupe*session, data=dataset2.dd.discri)
print(anova(dataset2.dd.discri.lm))

######### BEHAVIOR ########
##### LIM
GROUPE <- c("lim_T1", "lim_T2prime", "lim_T3prime", "lim_T4prime", "lim_T5prime")

for (groupe in GROUPE){
  print(paste0("Resultats pour ", groupe))
  dataset2.lim.groupe.hab <- dataset2.lim[(dataset2.lim$groupe==groupe &
(dataset2.lim$session=="Hab1"|
                                           dataset2.lim$session=="Hab2"|
                                           dataset2.lim$session=="Hab3"|
                                           dataset2.lim$session=="Hab4")),]
  dataset2.lim.groupe.hab <- droplevels(dataset2.lim.groupe.hab)
  dataset2.lim.groupe.hab.lme <- lme(temps~session,random=~1|souris,
data=dataset2.lim.groupe.hab);
  print(anova(dataset2.lim.groupe.hab.lme))

  dataset2.lim.groupe.discri <- dataset2.lim[(dataset2.lim$groupe==groupe &
(dataset2.lim$session=="Hab4"|
                                             dataset2.lim$session=="Test")),]
  #var.test(dataset2.lim.groupe.discri[dataset2.lim.groupe.discri$session=='Hab4',]$temps,
  #        dataset2.lim.groupe.discri[dataset2.lim.groupe.discri$session=='Test',]$temps)
  print(t.test(dataset2.lim.groupe.discri[dataset2.lim.groupe.discri$session=='Hab4',]$temps,
               dataset2.lim.groupe.discri[dataset2.lim.groupe.discri$session=='Test',]$temps,
               alternative = c("less"), var.equal = TRUE, paired = TRUE))
  readline(prompt="Press enter to continue")
}


##### DD
GROUPE2 <- c("dd_T1", "dd_T2prime", "dd_T3prime", "dd_T4prime", "dd_T5prime")
```

```
for (groupe in GROUPE2){
  print(paste0("Resultats pour ", groupe))
  dataset2.dd.groupe.hab <- dataset2.dd[(dataset2.dd$groupe==groupe &
(dataset2.dd$session=="Hab1"|
                                            dataset2.dd$session=="Hab2"|
                                            dataset2.dd$session=="Hab3"|
                                            dataset2.dd$session=="Hab4")),]
  dataset2.dd.groupe.hab <- droplevels(dataset2.dd.groupe.hab)
  dataset2.dd.groupe.hab.lme <- lme(temps~session,random=~1|souris,
data=dataset2.dd.groupe.hab);
  print(anova(dataset2.dd.groupe.hab.lme))
  print(summary(glht(dataset2.dd.groupe.hab.lme,linfct=mcp(session="Tukey"))))

  dataset2.dd.groupe.discri <- dataset2.dd[(dataset2.dd$groupe==groupe &
(dataset2.dd$session=="Hab4"|
                                              dataset2.dd$session=="Test")),]
  var.test(dataset2.dd.groupe.discri[dataset2.dd.groupe.discri$session=='Hab4',]$temps,
       dataset2.dd.groupe.discri[dataset2.dd.groupe.discri$session=='Test',]$temps)
  print(t.test(dataset2.dd.groupe.discri[dataset2.dd.groupe.discri$session=='Hab4',]$temps,
         dataset2.dd.groupe.discri[dataset2.dd.groupe.discri$session=='Test',]$temps,
         alternative = c("less"), var.equal = TRUE, paired = TRUE))
}

################################################################################
########################
##########################################    graphiques
##########################################
################################################################################
########################

### LIMONENE
infodata.dataset2.lim<-ddply(dataset2.lim,c("groupe", "session"),summarise,
                N = length(temps), mean=mean(temps), sd=sd(temps), sem=sd/sqrt(N))

graph.dataset2.lim.manip2<-ggplot((data=infodata.dataset2.lim[(infodata.dataset2.lim$session ==
"Hab4" | infodata.dataset2.lim$session == "Test"),]),
                 aes(x=groupe,y=mean, fill=session)) +
  geom_jitter(data = dataset2.lim[(dataset2.lim$session == "Hab4" | dataset2.lim$session ==
"Test"),],
       aes(x=groupe,y=temps),size=1.5,
       show.legend = FALSE, position = position_jitterdodge(jitter.width = 0.25, jitter.height = 0,
dodge.width = 0.6)) +
  geom_bar(stat="identity", show.legend=TRUE, position=position_dodge(width=0.6), colour =
"black", width=0.6, alpha=0.7)+
  geom_errorbar(aes(ymin=mean,ymax=mean+sem, width=0.3),
position=position_dodge(width=0.6))+
  scale_x_discrete(labels = c("T1", "T2'", "T3'", "T4'", "T5'"))+
  scale_fill_manual(values=c("grey80", "grey30"), label = c("Hab4", 'Test'))+
  scale_y_continuous("Investigation time (s)",
          limits=c(-0.001,4), breaks=c(0,1,2,3,4),
          expand = c(0,0))+
```

```
  labs(title="(+)limonene / (-)limonene")+
  theme(panel.background=element_rect(fill="white"),plot.title=element_text(size=18),
        axis.line.x=element_line(color = "black", size= .5), axis.line.y = element_line(color = "black", 
size = .5), axis.ticks=element_line(size=2),
        axis.title.y=element_text(size=22), axis.title.x=element_text(size=0),
        axis.text.x=element_text(size=18, colour="black"), axis.text.y=element_text(size=18, 
colour="black"),
        panel.grid.minor=element_blank(), panel.grid.major=element_blank(),
        legend.title=element_blank())
print(graph.dataset2.lim.manip2)
ggsave("D:/Recherches/These/Recherches/Manip apprentissage perceptif 
changeant/graph_dataset_lim_manip2.svg")

### DEC DODEC
infodata.dataset2.dd<-ddply(dataset2.dd,c("groupe", "session"),summarise,
                            N = length(temps), mean=mean(temps), sd=sd(temps), sem=sd/sqrt(N))

graph.dataset2.dd.manip2<-ggplot((data=infodata.dataset2.dd[(infodata.dataset2.dd$session == 
"Hab4" | infodata.dataset2.dd$session == "Test"),]),
                                 aes(x=groupe,y=mean, fill=session)) +
  geom_jitter(data = dataset2.dd[(dataset2.dd$session == "Hab4" | dataset2.dd$session == "Test"),],
              aes(x=groupe,y=temps),size=1.5,
              show.legend = FALSE, position = position_jitterdodge(jitter.width = 0.25, jitter.height = 0, 
dodge.width = 0.6)) +
  geom_bar(stat="identity", show.legend=TRUE, position=position_dodge(width=0.6), colour = 
"black", width=0.6, alpha=0.7)+
  geom_errorbar(aes(ymin=mean,ymax=mean+sem, width=0.3), 
position=position_dodge(width=0.6))+
  scale_x_discrete(labels = c("T1", "T2'", "T3'", "T4'", "T5'"))+
  scale_fill_manual(values=c("grey80", "grey30"), label = c("Hab4", 'Test'))+
  scale_y_continuous("Investigation time (s)",
                     limits=c(-0.001,4), breaks=c(0,1,2,3,4),
                     expand = c(0,0))+
  labs(title="decanal / dodecanone")+
  theme(panel.background=element_rect(fill="white"),plot.title=element_text(size=18),
        axis.line.x=element_line(color = "black", size= .5), axis.line.y = element_line(color = "black", 
size = .5), axis.ticks=element_line(size=2),
        axis.title.y=element_text(size=22), axis.title.x=element_text(size=0),
        axis.text.x=element_text(size=18, colour="black"), axis.text.y=element_text(size=18, 
colour="black"),
        panel.grid.minor=element_blank(), panel.grid.major=element_blank(),
        legend.title=element_blank())
print(graph.dataset2.dd.manip2)
ggsave("D:/Recherches/These/Recherches/Manip apprentissage perceptif 
changeant/graph_dataset_dd_manip2.svg")


########################################################################################
########################
########################################     graphiques habituation
########################################
```

```
################################################################################
########################

### LIMONENE
graph.dataset2.lim.manip2.habituation<-
ggplot((data=infodata.dataset2.lim[(infodata.dataset2.lim$session == "Hab1" |
                                   infodata.dataset2.lim$session == "Hab2" |
                                   infodata.dataset2.lim$session == "Hab3" |
                                   infodata.dataset2.lim$session == "Hab4"),]),
                          aes(x=groupe,y=mean, fill=session)) +
  geom_jitter(data = dataset2.lim[(dataset2.lim$session == "Hab1" |
                             dataset2.lim$session == "Hab2" |
                             dataset2.lim$session == "Hab3" |
                             dataset2.lim$session == "Hab4" ),],
            aes(x=groupe,y=temps),size=1.5,
            show.legend = FALSE, position = position_jitterdodge(jitter.width = 0.25, jitter.height = 0,
dodge.width = 0.6)) +
  geom_bar(stat="identity", show.legend=TRUE, position=position_dodge(width=0.6), colour =
"black", width=0.6, alpha=0.7)+
  geom_errorbar(aes(ymin=mean,ymax=mean+sem, width=0.3),
position=position_dodge(width=0.6))+
  scale_x_discrete(labels = c("T1", "T2'", "T3'", "T4'", "T5'"))+
  scale_fill_manual(values=c("grey50", "grey60", "grey70", "grey80"), label = c("OHab1", 'OHab2',
"OHab3", "OHab4"))+
  scale_y_continuous("Investigation time (s)",
                 limits=c(-0.001,5), breaks=c(0,1,2,3,4,5),
                 expand = c(0,0))+
  labs(title="(+)limonene / (-)limonene")+
  theme(panel.background=element_rect(fill="white"),plot.title=element_text(size=18),
       axis.line.x=element_line(color = "black", size= .5), axis.line.y = element_line(color = "black",
size = .5), axis.ticks=element_line(size=2),
       axis.title.y=element_text(size=22), axis.title.x=element_text(size=0),
       axis.text.x=element_text(size=18, colour="black"), axis.text.y=element_text(size=18,
colour="black"),
       panel.grid.minor=element_blank(), panel.grid.major=element_blank(),
       legend.title=element_blank())
print(graph.dataset2.lim.manip2.habituation)
#ggsave("D:/Jerem/Documents/Manip Lyon/perceptif
changeant/graph_dataset_lim_manip2_habituation.svg")
ggsave("N:/neuropop/J?r?my/J?r?my/Recherches/Th?se + Stage M2/Recherches/Manip
apprentissage perceptif changeant/graph_dataset_lim_manip2_habituation.svg")


### DEC DODEC
graph.dataset2.dd.manip2.habituation<-
ggplot((data=infodata.dataset2.dd[(infodata.dataset2.dd$session == "Hab1" |
                                  infodata.dataset2.dd$session == "Hab2" |
                                  infodata.dataset2.dd$session == "Hab3" |
                                  infodata.dataset2.dd$session == "Hab4"),]),
                         aes(x=groupe,y=mean, fill=session)) +
  geom_jitter(data = dataset2.dd[(dataset2.dd$session == "Hab1" |
                            dataset2.dd$session == "Hab2" |
```

```
                              dataset2.dd$session == "Hab3" |
                              dataset2.dd$session == "Hab4"),],
            aes(x=groupe,y=temps),size=1.5,
            show.legend = FALSE, position = position_jitterdodge(jitter.width = 0.25, jitter.height = 0,
dodge.width = 0.6)) +
  geom_bar(stat="identity", show.legend=TRUE, position=position_dodge(width=0.6), colour =
"black", width=0.6, alpha=0.7)+
  geom_errorbar(aes(ymin=mean,ymax=mean+sem, width=0.3),
position=position_dodge(width=0.6))+
  scale_x_discrete(labels = c("T1", "T2'", "T3'", "T4'", "T5'"))+
  scale_fill_manual(values=c("grey50", "grey60", "grey70", "grey80"), label = c("OHab1", 'OHab2',
"OHab3", "OHab4"))+
  scale_y_continuous("Investigation time (s)",
                limits=c(-0.001,5), breaks=c(0,1,2,3,4,5),
                expand = c(0,0))+
  labs(title="decanal / dodecanone")+
  theme(panel.background=element_rect(fill="white"),plot.title=element_text(size=18),
        axis.line.x=element_line(color = "black", size= .5), axis.line.y = element_line(color = "black",
size = .5), axis.ticks=element_line(size=2),
        axis.title.y=element_text(size=22), axis.title.x=element_text(size=0),
        axis.text.x=element_text(size=18, colour="black"), axis.text.y=element_text(size=18,
colour="black"),
        panel.grid.minor=element_blank(), panel.grid.major=element_blank(),
        legend.title=element_blank())
print(graph.dataset2.dd.manip2.habituation)
#ggsave("D:/Jerem/Documents/Manip Lyon/perceptif
changeant/graph_dataset_dd_manip2_habituation.svg")
ggsave("N:/neuropop/J?r?my/J?r?my/Recherches/Th?se + Stage M2/Recherches/Manip
apprentissage perceptif changeant/graph_dataset_dd_manip2_habituation.svg")



########    BRDU     ########
dataset.brdu.manip2 <- read.csv("brdu_prime.csv", header=T, sep = ",", dec = ",")
dataset.brdu.manip2$densite <- dataset.brdu.manip2$densite*100000

dataset.brdu.manip1.T1 <-dataset.brdu.manip1[dataset.brdu.manip1$group == "T1",
c("X","animal", "densite", "groupe", "group" )]
dataset.brdu.manip1.T1$densite <- dataset.brdu.manip1.T1$densite*100000
dataset.brdu.manip2 <- rbind(dataset.brdu.manip2, dataset.brdu.manip1.T1)

dataset.brdu.manip2.lm <- lm(densite~group, data=dataset.brdu.manip2)
print(anova(dataset.brdu.manip2.lm))
print(summary(glht(dataset.brdu.manip2.lm,linfct=mcp(group="Tukey"))))
print(pairwise.t.test(dataset.brdu.manip2$densite, dataset.brdu.manip2$group, var.equal=TRUE,
p.adjust = "holm"))

## GROUPED ACCORDING TO GROUPS
infodata.brdu.manip2 <- ddply(dataset.brdu.manip2,c("group"),summarise,
                     N = length(densite), mean=mean(densite), sd=sd(densite), sem=sd/sqrt(N))
```

```
infodata.brdu.manip2$group <- factor(infodata.brdu.manip2$group, levels=c("T1", "T2prime",
"T3prime", "T4prime", "T5prime"))
dataset.brdu.manip2$group <- factor(dataset.brdu.manip2$group, levels=c("T1", "T2prime",
"T3prime", "T4prime", "T5prime"))

graph.dataset.brdu.manip2<-ggplot((data=infodata.brdu.manip2),aes(x=group,y=mean, fill=group))
+
  geom_jitter(data = dataset.brdu.manip2,
            aes(x=group,y=densite),size=1.5, show.legend = FALSE,
            position = position_jitterdodge(jitter.width = 0.25, jitter.height = 0, dodge.width = 0.6)) +
  geom_bar(stat="identity", show.legend=FALSE, position=position_dodge(width=0.6), colour =
"black", width=0.6, alpha=0.7)+
  geom_errorbar(aes(ymin=mean,ymax=mean+sem, width=0.3),
position=position_dodge(width=0.6))+
  scale_x_discrete(labels = c("T1", "T2'", "T3'", "T4'", "T5'"))+
  scale_fill_manual(values=c("grey90", "grey30", "grey30", "grey30", "grey30"))+
  scale_y_continuous("Brdu-positive cell density (cell/?m? *" ~ 10^-05 ~")",
                  limits=c(-0.0001,9), breaks=c(0,1,2,3,4,5,6,7,8,9),
                  expand = c(0,0))+
  labs(title="")+
  theme(panel.background=element_rect(fill="white"),plot.title=element_text(size=18),
        axis.line.x=element_line(color = "black", size= .5), axis.line.y = element_line(color = "black",
size = .5), axis.ticks=element_line(size=2),
        axis.title.y=element_text(size=22), axis.title.x=element_text(size=0),
        axis.text.x=element_text(angle=0, hjust=0.5,size=18, colour="black"),
axis.text.y=element_text(size=18, colour="black"),
        panel.grid.minor=element_blank(), panel.grid.major=element_blank(),
        legend.title=element_blank())
print(graph.dataset.brdu.manip2)
#ggsave("D:/Jerem/Documents/Manip Lyon/perceptif changeant/graph_dataset_brdu_manip2.svg",
width=7, height=8)
ggsave("N:/neuropop/J?r?my/J?r?my/Recherches/Th?se + Stage M2/Recherches/Manip
apprentissage perceptif changeant/graph_dataset_brdu_manip2.pdf")



## Correlation index discri et brdu densite
dataset2.lim.discri$index <- 1-(dataset2.lim.discri$Hab4 / dataset2.lim.discri$Test)  ## reprendre
dataset en large (en haut)
for (i in seq(dataset2.lim.discri$index)){
  if (is.nan(dataset2.lim.discri$index[i])){
    dataset2.lim.discri$index[i] <- 0
  }
  else if(dataset2.lim.discri$index[i] == -Inf){
    dataset2.lim.discri$index[i] <- 0
  }
  else if (dataset2.lim.discri$index[i] < 0){
    dataset2.lim.discri$index[i] <- 0
  }
  else {
    dataset2.lim.discri$index[i] <- dataset2.lim.discri$index[i]
  }
```

```
}

dataset2.lim.discri.summary<-ddply(dataset2.lim.discri,c("groupe"),summarise,
                    N = length(index), mean=mean(index), sd=sd(index), sem=sd/sqrt(N))
dataset.brdu.manip2.summary <- ddply(dataset.brdu.manip2,c("group"),summarise,
                     N = length(densite), mean=mean(densite), sd=sd(densite), sem=sd/sqrt(N))
dataset2.correlation <- data.frame(index =
c((dataset2.lim.discri.summary[dataset2.lim.discri.summary$groupe == 'lim_T1',]$mean),
                         (dataset2.lim.discri.summary[dataset2.lim.discri.summary$groupe ==
'lim_T2prime',]$mean),
                         (dataset2.lim.discri.summary[dataset2.lim.discri.summary$groupe ==
'lim_T3prime',]$mean),
                         (dataset2.lim.discri.summary[dataset2.lim.discri.summary$groupe ==
'lim_T4prime',]$mean),
                         (dataset2.lim.discri.summary[dataset2.lim.discri.summary$groupe ==
'lim_T5prime',]$mean)),
                    densite =
c((dataset.brdu.manip2.summary[dataset.brdu.manip2.summary$group == 'T1',]$mean),
                          (dataset.brdu.manip2.summary[dataset.brdu.manip2.summary$group
== 'T2prime',]$mean),
                          (dataset.brdu.manip2.summary[dataset.brdu.manip2.summary$group
== 'T3prime',]$mean),
                          (dataset.brdu.manip2.summary[dataset.brdu.manip2.summary$group
== 'T4prime',]$mean),
                          (dataset.brdu.manip2.summary[dataset.brdu.manip2.summary$group
== 'T5prime',]$mean)))

print(cor.test(dataset2.correlation$index, dataset2.correlation$densite))





## ## ## ## ## ## ## ## ## ## ## ## ## ## ## ## ## ## ## ##
## GRAPH WITH COMPLEMENTARY DATA FOR REVIEWERS ### ## ## ##
## ## ## ## ## ## ## ## ## ## ## ## ## ## ## ## ## ## ## ##
getwd()
setwd('../new_data_for_review/')
list.files()



###Figure 1Ci
dataset.fig1Ci <- read.csv("figure 1Ci.csv", header=T, sep = ",", dec = ",")

infodata.dataset.fig1Ci <- ddply(dataset.fig1Ci,c("subgrroup", "group"),summarise,
                   N = length(densite), mean=mean(densite), sd=sd(densite), sem=sd/sqrt(N))

infodata.dataset.fig1Ci$group  <- c("T1", "T2", "T3", "T4", "T5")
dataset.fig1Ci$group  <- c("T1", "T1","T1","T1",
                "T2", "T2","T2","T2",
```

```
                        "T3", "T3","T3","T3",
                        "T4","T4","T4","T4",
                        "T5", "T5", "T5", "T5",
                        "T1", "T1",
                        "T2", "T2","T2","T2","T2",
                        "T3", "T3",
                        "T4","T4","T4",
                        "T5", "T5", "T5", "T5")
dataset.fig1Ci$group <- factor(dataset.fig1Ci$group, levels=c("T1NE", "T1", "T2", "T3", "T4",
"T5"))
dataset.fig1Ci$subgrroup <-factor(dataset.fig1Ci$subgrroup, levels=c("NE", "Enr"))
infodata.dataset.fig1Ci$subgrroup <-factor(infodata.dataset.fig1Ci$subgrroup, levels=c("NE",
"Enr"))

graph.dataset.fig1Ci<-ggplot((data=infodata.dataset.fig1Ci),aes(x=group,y=mean, fill=subgrroup))
+
  geom_jitter(data = dataset.fig1Ci,
            aes(x=group,y=densite, fill=subgrroup),size=1.5, show.legend = FALSE,
            position = position_jitterdodge(jitter.width = 0.25, jitter.height = 0, dodge.width = 0.6)) +
  geom_bar(stat="identity", show.legend=TRUE, position=position_dodge(width=0.6), colour =
"black", width=0.6, alpha=0.7)+
  geom_errorbar(aes(ymin=mean,ymax=mean+sem, width=0.3),
position=position_dodge(width=0.6))+
  scale_x_discrete(labels = c("T1", "T2", "T3", "T4", "T5"))+
  scale_fill_manual(values=c("grey30", "grey90"))+
  scale_y_continuous("Brdu-positive cell density (cell / mm²)",
                 limits=c(-0.0001,75), breaks=c(0,10,20,30,40,50,60,70),
                 expand = c(0,0))+
  labs(title="")+
  theme(panel.background=element_rect(fill="white"),plot.title=element_text(size=18),
      axis.line.x=element_line(color = "black", size= .5), axis.line.y = element_line(color = "black",
size = .5), axis.ticks=element_line(size=2),
      axis.title.y=element_text(size=22), axis.title.x=element_text(size=0),
      axis.text.x=element_text(angle=0, hjust=0.5,size=18, colour="black"),
axis.text.y=element_text(size=18, colour="black"),
      panel.grid.minor=element_blank(), panel.grid.major=element_blank(),
      legend.title=element_blank())
print(graph.dataset.fig1Ci)
ggsave("/media/jeremy/Data/CloudStation/temporaire/papier
changeant/new_data_for_review/graph_fig1Ci.pdf")

### figure 1D
dataset.fig1D <- read.csv("figure_1D_densite.csv", header=T, sep = ",", dec = ",")
colnames(dataset.fig1D) <- c("subgroup","group", "densite")

infodata.dataset.fig1D <- ddply(dataset.fig1D,c("subgroup", "group"),summarise,
                          N = length(densite), mean=mean(densite), sd=sd(densite), sem=sd/sqrt(N))

infodata.dataset.fig1D$group  <- c("T1", "T2", "T3", "T4", "T5",
                           "T1", "T2", "T3", "T4", "T5",
                           "T1", "T2", "T3", "T4", "T5")
infodata.dataset.fig1D$subgroup <- c('NE', 'NE','NE', 'NE', 'NE',
```

```
                              'lim', 'lim', 'lim', 'lim', 'lim',
                              'dec', 'dec', 'dec', 'dec','dec')
dataset.fig1D$group  <- c("T1", "T1",
                    "T2", "T2","T2","T2",
                    "T3", "T3",
                    "T4","T4","T4",
                    "T5", "T5", "T5", "T5",
                    "T1", "T1", "T1", "T1",
                    "T2", "T2","T2","T2",
                    "T3", "T3","T3","T3",
                    "T4","T4","T4","T4",
                    "T5", "T5", "T5", "T5",
                    "T1", "T1","T1", "T1",
                    "T2", "T2","T2","T2",
                    "T3", "T3","T3","T3",
                    "T4","T4","T4","T4",
                    "T5", "T5", "T5", "T5")
dataset.fig1D$subgroup <- c("NE", "NE","NE","NE","NE","NE","NE","NE","NE","NE",
                     "NE","NE","NE","NE","NE",
                     "lim", "lim","lim","lim","lim","lim","lim","lim","lim","lim",
                     "lim","lim","lim","lim","lim","lim","lim","lim","lim","lim",
                     "dec", "dec","dec","dec","dec","dec","dec","dec","dec","dec",
                     "dec","dec","dec","dec","dec","dec","dec","dec","dec","dec")

dataset.fig1D$group <- factor(dataset.fig1D$group, levels=c("T1", "T2", "T3", "T4", "T5"))
dataset.fig1D$subgroup <- factor(dataset.fig1D$subgroup, levels=c("NE",
                                                    "lim",
                                                    "dec"))
infodata.dataset.fig1D$group <- factor(infodata.dataset.fig1D$group, levels=c("T1", "T2", "T3",
"T4", "T5"))
infodata.dataset.fig1D$subgroup <- factor(infodata.dataset.fig1D$subgroup, levels=c("NE",
                                                                "lim",
                                                                "dec"))



graph.dataset.fig1D<-ggplot((data=infodata.dataset.fig1D),aes(x=group,y=mean, fill=subgroup)) +
  geom_jitter(data = dataset.fig1D,
           aes(x=group,y=densite, fill=subgroup),size=1.5, show.legend = FALSE,
           position = position_jitterdodge(jitter.width = 0.25, jitter.height = 0, dodge.width = 0.6)) +
  geom_bar(stat="identity", show.legend=TRUE, position=position_dodge(width=0.6), colour =
"black", width=0.6, alpha=0.7)+
  geom_errorbar(aes(ymin=mean,ymax=mean+sem, width=0.3),
position=position_dodge(width=0.6))+
  scale_x_discrete(labels = c("T1", "T2", "T3", "T4", "T5"))+
  scale_fill_manual(values=c("grey30", "grey50", "grey90"))+
  scale_y_continuous("Brdu-Zif268 -positive cell density \n (cell/mm²)",
                limits=c(0,25), breaks=c(0,5,10,15,20,25),
                expand = c(0,0))+
  labs(title="")+
  theme(panel.background=element_rect(fill="white"),plot.title=element_text(size=18),
```

```
        axis.line.x=element_line(color = "black", size= .5), axis.line.y = element_line(color = "black",
size = .5), axis.ticks=element_line(size=2),
        axis.title.y=element_text(size=22), axis.title.x=element_text(size=0),
        axis.text.x=element_text(angle=0, hjust=0.5,size=18, colour="black"),
axis.text.y=element_text(size=18, colour="black"),
        panel.grid.minor=element_blank(), panel.grid.major=element_blank(),
        legend.title=element_blank(), legend.position="right")
print(graph.dataset.fig1D)
ggsave("/media/jeremy/Data/CloudStation/temporaire/papier
changeant/new_data_for_review/graph_fig1D.pdf")

### figure 2D
dataset.fig2D <- read.csv("figure 2D_densite.csv", header=T, sep = ",", dec = ",")
colnames(dataset.fig2D) <- c("subgroup","group", "densite")

infodata.dataset.fig2D <- ddply(dataset.fig2D,c("subgroup", "group"),summarise,
                                N = length(densite), mean=mean(densite), sd=sd(densite), sem=sd/sqrt(N))

infodata.dataset.fig2D$group  <- c("T1", "T2", "T3", "T4", "T5",
                                   "T1", "T2", "T3", "T4", "T5")
infodata.dataset.fig2D$subgroup <- c('Lim', 'Lim',
                                     'Lim', 'Lim',
                                     'Lim',
                                     'Dec', 'Dec', 'Dec',
                                     'Dec', 'Dec')
dataset.fig2D$group  <- c("T1", "T1","T1", "T1",
                          "T2", "T2","T2","T2","T2", "T2",
                          "T3", "T3","T3", "T3",
                          "T4","T4","T4","T4","T4","T4",
                          "T5", "T5", "T5", "T5","T5", "T5",
                          "T1", "T1", "T1", "T1",
                          "T2", "T2","T2","T2", "T2","T2",
                          "T3", "T3","T3","T3",
                          "T4","T4","T4","T4","T4","T4",
                          "T5", "T5", "T5", "T5","T5", "T5")
dataset.fig2D$subgroup <- c("Lim", "Lim","Lim","Lim","Lim","Lim","Lim","Lim","Lim","Lim",
                            "Lim","Lim","Lim","Lim","Lim","Lim","Lim","Lim","Lim","Lim",
                            "Lim","Lim","Lim","Lim","Lim","Lim",
                            "Dec", "Dec","Dec","Dec","Dec","Dec","Dec","Dec","Dec","Dec",
                            "Dec","Dec","Dec","Dec","Dec","Dec","Dec","Dec","Dec","Dec",
                            "Dec","Dec","Dec","Dec","Dec","Dec")

dataset.fig2D$group <- factor(dataset.fig2D$group, levels=c("T1", "T2", "T3", "T4", "T5"))
dataset.fig2D$subgroup <- factor(dataset.fig2D$subgroup, levels=c("Lim",
                                                                  "Dec"))
infodata.dataset.fig2D$group <- factor(infodata.dataset.fig2D$group, levels=c("T1", "T2", "T3",
"T4", "T5"))
infodata.dataset.fig2D$subgroup <- factor(infodata.dataset.fig2D$subgroup, levels=c("Lim",
                                                                                    "Dec"))
```

```
graph.dataset.fig2D<-ggplot((data=infodata.dataset.fig2D),aes(x=group,y=mean, fill=subgroup)) +
  geom_jitter(data = dataset.fig2D,
              aes(x=group,y=densite, fill=subgroup),size=1.5, show.legend = FALSE,
              position = position_jitterdodge(jitter.width = 0.25, jitter.height = 0, dodge.width = 0.6)) +
  geom_bar(stat="identity", show.legend=TRUE, position=position_dodge(width=0.6), colour =
"black", width=0.6, alpha=0.7)+
  geom_errorbar(aes(ymin=mean,ymax=mean+sem, width=0.3),
position=position_dodge(width=0.6))+
  scale_x_discrete(labels = c("T1", "T2", "T3", "T4", "T5"))+
  scale_fill_manual(values=c("grey50", "grey90"))+
  scale_y_continuous("Brdu-Zif268 -positive cell density \n (cell/mm²)",
                     limits=c(-0.0001,30), breaks=c(0,5,10,15,20,25,30),
                     expand = c(0,0))+
  labs(title="")+
  theme(panel.background=element_rect(fill="white"),plot.title=element_text(size=18),
        axis.line.x=element_line(color = "black", size= .5), axis.line.y = element_line(color = "black",
size = .5), axis.ticks=element_line(size=2),
        axis.title.y=element_text(size=22), axis.title.x=element_text(size=0),
        axis.text.x=element_text(angle=0, hjust=0.5,size=18, colour="black"),
axis.text.y=element_text(size=18, colour="black"),
        panel.grid.minor=element_blank(), panel.grid.major=element_blank(),
        legend.title=element_blank(), legend.position="right")
print(graph.dataset.fig2D)
ggsave("/media/jeremy/Data/CloudStation/temporaire/papier
changeant/new_data_for_review/graph_fig2D.pdf")




######### ######### ######### #########
######### FIGURE 3 ###################
######### ######### ##################
rm(list = ls())

##### Library ######
library(xlsx)
library(ggplot2)
library(plyr)
library(reshape2)
library(nlme)
library(multcomp)

setwd("D:/Jerem/Documents/Manip Lyon/perceptif changeant/data_csv")
setwd("/media/jeremy/Data/data_csv")

##################################################################
#################    2mois IDU CLDU  #########################
######   Reading datasets behavior and idu/cldu density ######
#################################################################
```

```
###LIM BEHAVIOR
dataset.idu.cldu.groupe1.lim <- read.csv("dataset_idu_cldu_groupe1_lim.csv", header=T, sep = ",")
dataset.idu.cldu.groupe1.lim$groupe <-  "groupe1"

#Groupe 2 est meme protocol que T2prime de manip 2 donc concatenation des 2
dataset.idu.cldu.groupe2.lim_1 <- read.csv("dataset_idu_cldu_groupe2_lim.csv", header=T, sep =
",")
dataset.idu.cldu.groupe2.lim_1$groupe <-  "groupe2"
dataset.idu.cldu.groupe2.lim_2 <- read.csv("dataset_lim_T2_prime.csv", header=T, sep = ",")
dataset.idu.cldu.groupe2.lim_2$groupe <-  "groupe2"
dataset.idu.cldu.groupe2.lim <- rbind(dataset.idu.cldu.groupe2.lim_1,
dataset.idu.cldu.groupe2.lim_2)

dataset.idu.cldu.groupe3.lim <- read.csv("dataset_idu_cldu_groupe3_lim.csv", header=T, sep = ",")
dataset.idu.cldu.groupe3.lim$groupe <-  "groupe3"

#Groupe 4 est meme protocol que T3prime de manip 2 donc concatenation des 2
dataset.idu.cldu.groupe4.lim_1 <- read.csv("dataset_idu_cldu_groupe4_lim.csv", header=T, sep =
",")
dataset.idu.cldu.groupe4.lim_1$groupe <- "groupe4"
dataset.idu.cldu.groupe4.lim_2 <- read.csv("dataset_lim_T3_prime.csv", header=T, sep = ",")
dataset.idu.cldu.groupe4.lim_2$groupe <- "groupe4"
dataset.idu.cldu.groupe4.lim <- rbind(dataset.idu.cldu.groupe4.lim_1,
dataset.idu.cldu.groupe4.lim_2)

## DD
dataset.idu.cldu.groupe1.dd <- read.csv("dataset_idu_cldu_groupe1_dd.csv", header=T, sep = ",")
dataset.idu.cldu.groupe1.dd$groupe <-  "groupe1"

dataset.idu.cldu.groupe2.dd_1 <- read.csv("dataset_idu_cldu_groupe2_dd.csv", header=T, sep = ",")
dataset.idu.cldu.groupe2.dd_1$groupe <-  "groupe2"
dataset.idu.cldu.groupe2.dd_2 <- read.csv("dataset_dd_T2_prime.csv", header=T, sep = ",")
dataset.idu.cldu.groupe2.dd_2$groupe <-  "groupe2"
dataset.idu.cldu.groupe2.dd <- rbind(dataset.idu.cldu.groupe2.dd_1, dataset.idu.cldu.groupe2.dd_2)

dataset.idu.cldu.groupe3.dd <- read.csv("dataset_idu_cldu_groupe3_dd.csv", header=T, sep = ",")
dataset.idu.cldu.groupe3.dd$groupe <-  "groupe3"

dataset.idu.cldu.groupe4.dd_1 <- read.csv("dataset_idu_cldu_groupe4_dd.csv", header=T, sep = ",")
dataset.idu.cldu.groupe4.dd_1$groupe <- "groupe4"
dataset.idu.cldu.groupe4.dd_2 <- read.csv("dataset_dd_T3_prime.csv", header=T, sep = ",")
dataset.idu.cldu.groupe4.dd_2$groupe <- "groupe4"
dataset.idu.cldu.groupe4.dd <- rbind(dataset.idu.cldu.groupe4.dd_1, dataset.idu.cldu.groupe4.dd_2)



dataset.lim <- rbind.fill(dataset.idu.cldu.groupe1.lim, dataset.idu.cldu.groupe2.lim,
                    dataset.idu.cldu.groupe3.lim, dataset.idu.cldu.groupe4.lim)
dataset.lim <- na.omit(dataset.lim)
dataset.lim <- dataset.lim[, c("Hab1","Hab2", "Hab3", "Hab4", "Test", "groupe", "X")]
dataset.lim.discri <- dataset.lim
```

```
dataset.lim <- melt(dataset.lim,
measure.vars=c("Hab1","Hab2","Hab3","Hab4","Test"),factorAsStrings=F)
names(dataset.lim)[2]<-"souris"
names(dataset.lim)[3]<-"session"
names(dataset.lim)[4]<-"temps"



dataset.dd <- rbind.fill(dataset.idu.cldu.groupe1.dd, dataset.idu.cldu.groupe2.dd,
                         dataset.idu.cldu.groupe3.dd, dataset.idu.cldu.groupe4.dd)
dataset.dd <- na.omit(dataset.dd)
dataset.dd <- dataset.dd[, c("Hab1","Hab2", "Hab3", "Hab4", "Test", "groupe", "X")]
dataset.dd.discri <- dataset.dd
dataset.dd <- melt(dataset.dd,
measure.vars=c("Hab1","Hab2","Hab3","Hab4","Test"),factorAsStrings=F)
names(dataset.dd)[2]<-"souris"
names(dataset.dd)[3]<-"session"
names(dataset.dd)[4]<-"temps"


###################################################################################
##########################
############################################     statistiques
############################################
###################################################################################
##########################
dataset.lim.discri <- dataset.lim[(dataset.lim$session == 'Hab4'|dataset.lim$session == 'Test'),]
dataset.lim.discri.lm <- lm(temps~session*groupe, data=dataset.lim.discri)
print(anova(dataset.lim.discri.lm))

dataset.dd.discri <- dataset.dd[(dataset.dd$session == 'Hab4'|dataset.dd$session == 'Test'),]
dataset.dd.discri.lm <- lm(temps~groupe*session, data=dataset.dd.discri)
print(anova(dataset.dd.discri.lm))



##### LIM
GROUPE <- c("groupe1", "groupe2", "groupe3", "groupe4")

for (groupe in GROUPE){
  print(paste0("Resultats pour ", groupe))
  dataset.lim.groupe.hab <- dataset.lim[(dataset.lim$groupe==groupe &
(dataset.lim$session=="Hab1"|
                                        dataset.lim$session=="Hab2"|
                                        dataset.lim$session=="Hab3"|
                                        dataset.lim$session=="Hab4")),]
  dataset.lim.groupe.hab <- droplevels(dataset.lim.groupe.hab)
  dataset.lim.groupe.hab.lme <- lme(temps~session,random=~1|souris,
data=dataset.lim.groupe.hab);
  print(anova(dataset.lim.groupe.hab.lme))
  print(summary(glht(dataset.lim.groupe.hab.lme,linfct=mcp(session="Tukey"))))
```

```
  dataset.lim.groupe.discri <- dataset.lim[(dataset.lim$groupe==groupe &
(dataset.lim$session=="Hab4"|
                                                          dataset.lim$session=="Test")),]
  var.test(dataset.lim.groupe.discri[dataset.lim.groupe.discri$session=='Hab4',]$temps,
         dataset.lim.groupe.discri[dataset.lim.groupe.discri$session=='Test',]$temps)
  print(t.test(dataset.lim.groupe.discri[dataset.lim.groupe.discri$session=='Hab4',]$temps,
             dataset.lim.groupe.discri[dataset.lim.groupe.discri$session=='Test',]$temps,
             alternative = c("less"), var.equal = TRUE, paired = TRUE))
}



##### DD
GROUPE2 <- c("groupe1", "groupe2", "groupe3", "groupe4")

for (groupe in GROUPE){
  print(paste0("Resultats pour ", groupe))
  dataset.dd.groupe.hab <- dataset.dd[(dataset.dd$groupe==groupe & (dataset.dd$session=="Hab1"|
                                                      dataset.dd$session=="Hab2"|
                                                      dataset.dd$session=="Hab3"|
                                                      dataset.dd$session=="Hab4")),]
  dataset.dd.groupe.hab <- droplevels(dataset.dd.groupe.hab)
  dataset.dd.groupe.hab.lme <- lme(temps~session,random=~1|souris, data=dataset.dd.groupe.hab);
  print(anova(dataset.dd.groupe.hab.lme))
  print(summary(glht(dataset.dd.groupe.hab.lme,linfct=mcp(session="Tukey"))))

  dataset.dd.groupe.discri <- dataset.dd[(dataset.dd$groupe==groupe &
(dataset.dd$session=="Hab4"|
                                                        dataset.dd$session=="Test")),]
  var.test(dataset.dd.groupe.discri[dataset.dd.groupe.discri$session=='Hab4',]$temps,
         dataset.dd.groupe.discri[dataset.dd.groupe.discri$session=='Test',]$temps)
  print(t.test(dataset.dd.groupe.discri[dataset.dd.groupe.discri$session=='Hab4',]$temps,
             dataset.dd.groupe.discri[dataset.dd.groupe.discri$session=='Test',]$temps,
             alternative = c("less"), var.equal = TRUE, paired = TRUE))
}


##################################################################################
#########################
###########################################    graphiques
###########################################
##################################################################################
#########################

### LIMONENE
infodata.dataset.lim<-ddply(dataset.lim,c("groupe", "session"),summarise,
                        N = length(temps), mean=mean(temps), sd=sd(temps), sem=sd/sqrt(N))

graph.dataset.lim.manip<-ggplot((data=infodata.dataset.lim[(infodata.dataset.lim$session ==
"Hab4" | infodata.dataset.lim$session == "Test"),]),
                          aes(x=groupe,y=mean, fill=session)) +
  geom_jitter(data = dataset.lim[(dataset.lim$session == "Hab4" | dataset.lim$session == "Test"),],
```

```
            aes(x=groupe,y=temps),size=1.5,
            show.legend = FALSE, position = position_jitterdodge(jitter.width = 0.25, jitter.height = 0, 
dodge.width = 0.6)) +
  geom_bar(stat="identity", show.legend=TRUE, position=position_dodge(width=0.6), colour = 
"black", width=0.6, alpha=0.7)+
  geom_errorbar(aes(ymin=mean,ymax=mean+sem, width=0.3), 
position=position_dodge(width=0.6))+
  scale_x_discrete(labels = c("Groupe 1", "Groupe 2", "Groupe 3", "Groupe 4"))+
  scale_fill_manual(values=c("grey80", "grey30"), label = c("Hab4", 'Test'))+
  scale_y_continuous("Investigation time (s)", 
                  limits=c(-0.001,8), breaks=c(0,1,2,3,4,5,6,7,8), 
                  expand = c(0,0))+
  labs(title="(+)limonene / (-)limonene")+
  theme(panel.background=element_rect(fill="white"),plot.title=element_text(size=18),
       axis.line.x=element_line(color = "black", size= .5), axis.line.y = element_line(color = "black", 
size = .5), axis.ticks=element_line(size=2),
       axis.title.y=element_text(size=22), axis.title.x=element_text(size=0), 
       axis.text.x=element_text(angle=35, hjust=1, size=18, colour="black"), 
axis.text.y=element_text(size=18, colour="black"),
       panel.grid.minor=element_blank(), panel.grid.major=element_blank(),
       legend.title=element_blank())
print(graph.dataset.lim.manip)
ggsave("D:/Jerem/Documents/Manip Lyon/perceptif 
changeant/graph_dataset_lim_manipIduCldu_2mois.svg")

###hab
graph.dataset.lim.manip.hab<-ggplot((data=infodata.dataset.lim[(infodata.dataset.lim$session == 
"Hab1" | 
                                                      infodata.dataset.lim$session == "Hab2" | 
                                                      infodata.dataset.lim$session == "Hab3" | 
                                                      infodata.dataset.lim$session == "Hab4"),]), 
                              aes(x=groupe,y=mean, fill=session)) +
  geom_jitter(data = dataset.lim[(dataset.lim$session == "Hab1" | dataset.lim$session == "Hab2" | 
                              dataset.lim$session == "Hab3" |dataset.lim$session == "Hab4"),], 
            aes(x=groupe,y=temps),size=1.5,
            show.legend = FALSE, position = position_jitterdodge(jitter.width = 0.25, jitter.height = 0, 
dodge.width = 0.6)) +
  geom_bar(stat="identity", show.legend=TRUE, position=position_dodge(width=0.6), colour = 
"black", width=0.6, alpha=0.7)+
  geom_errorbar(aes(ymin=mean,ymax=mean+sem, width=0.3), 
position=position_dodge(width=0.6))+
  #scale_x_discrete(labels = c("Groupe 1", "Groupe 2", "Groupe 3", "Groupe 4"))+
  scale_fill_manual(values=c("grey50", "grey60", "grey70", "grey80"), label = 
c("Hab1","Hab2","Hab3","Hab4"))+
  scale_y_continuous("Investigation time (s)", 
                  limits=c(-0.001,8), breaks=c(0,1,2,3,4,5,6,7,8), 
                  expand = c(0,0))+
  labs(title="(+)limonene / (-)limonene")+
  theme(panel.background=element_rect(fill="white"),plot.title=element_text(size=18),
       axis.line.x=element_line(color = "black", size= .5), axis.line.y = element_line(color = "black", 
size = .5), axis.ticks=element_line(size=2),
       axis.title.y=element_text(size=22), axis.title.x=element_text(size=0),
```

```
        axis.text.x=element_text(angle=35, hjust=1, size=18, colour="black"),
axis.text.y=element_text(size=18, colour="black"),
        panel.grid.minor=element_blank(), panel.grid.major=element_blank(),
        legend.title=element_blank())
print(graph.dataset.lim.manip.hab)
ggsave("D:/Jerem/Documents/Manip Lyon/perceptif
changeant/graph_dataset_lim_manipIduCldu_2mois_habituation.svg")



### DEC DODEC
infodata.dataset.dd<-ddply(dataset.dd,c("groupe", "session"),summarise,
                           N = length(temps), mean=mean(temps), sd=sd(temps), sem=sd/sqrt(N))

graph.dataset.dd.manip<-ggplot((data=infodata.dataset.dd[(infodata.dataset.dd$session == "Hab4" |
infodata.dataset.dd$session == "Test"),]),
                               aes(x=groupe,y=mean, fill=session)) +
  geom_jitter(data = dataset.dd[(dataset.dd$session == "Hab4" | dataset.dd$session == "Test"),],
              aes(x=groupe,y=temps),size=1.5,
              show.legend = FALSE, position = position_jitterdodge(jitter.width = 0.25, jitter.height = 0,
dodge.width = 0.6)) +
  geom_bar(stat="identity", show.legend=TRUE, position=position_dodge(width=0.6), colour =
"black", width=0.6, alpha=0.7)+
  geom_errorbar(aes(ymin=mean,ymax=mean+sem, width=0.3),
position=position_dodge(width=0.6))+
  scale_x_discrete(labels = c("Groupe 1", "Groupe 2", "Groupe 3", "Groupe 4"))+
  scale_fill_manual(values=c("grey80", "grey30"), label = c("Hab4", 'Test'))+
  scale_y_continuous("Investigation time (s)",
                     limits=c(-0.001,8), breaks=c(0,1,2,3,4,5,6,7,8),
                     expand = c(0,0))+
  labs(title="decanal / dodecanone")+
  theme(panel.background=element_rect(fill="white"),plot.title=element_text(size=18),
        axis.line.x=element_line(color = "black", size= .5), axis.line.y = element_line(color = "black",
size = .5), axis.ticks=element_line(size=2),
        axis.title.y=element_text(size=22), axis.title.x=element_text(size=0),
        axis.text.x=element_text(angle=35, hjust=1,size=18, colour="black"),
axis.text.y=element_text(size=18, colour="black"),
        panel.grid.minor=element_blank(), panel.grid.major=element_blank(),
        legend.title=element_blank())
print(graph.dataset.dd.manip)
ggsave("D:/Jerem/Documents/Manip Lyon/perceptif
changeant/graph_dataset_dd_manipIduCldu_2mois.svg")

##Behav
graph.dataset.dd.manip.hab<-ggplot((data=infodata.dataset.dd[(infodata.dataset.dd$session ==
"Hab1" | infodata.dataset.dd$session == "Hab2"
                                                              | infodata.dataset.dd$session == "Hab3" |
infodata.dataset.dd$session == "Hab4"),]),
                                   aes(x=groupe,y=mean, fill=session)) +
  geom_jitter(data = dataset.dd[(dataset.dd$session == "Hab1" | dataset.dd$session == "Hab2"|
                                   dataset.dd$session == "Hab3" |dataset.dd$session == "Hab4" ),],
              aes(x=groupe,y=temps),size=1.5,
```

```
            show.legend = FALSE, position = position_jitterdodge(jitter.width = 0.25, jitter.height = 0,
dodge.width = 0.6)) +
  geom_bar(stat="identity", show.legend=TRUE, position=position_dodge(width=0.6), colour =
"black", width=0.6, alpha=0.7)+
  geom_errorbar(aes(ymin=mean,ymax=mean+sem, width=0.3),
position=position_dodge(width=0.6))+
  scale_x_discrete(labels = c("Groupe 1", "Groupe 2", "Groupe 3", "Groupe 4"))+
  scale_fill_manual(values=c("grey50", "grey60", "grey70", "grey80"), label = c("Hab1", 'Hab2',
'Hab3', 'Hab4'))+
  scale_y_continuous("Investigation time (s)",
                 limits=c(-0.001,10), breaks=c(0,1,2,3,4,5,6,7,8,9,10),
                 expand = c(0,0))+
  labs(title="decanal / dodecanone")+
  theme(panel.background=element_rect(fill="white"),plot.title=element_text(size=18),
      axis.line.x=element_line(color = "black", size= .5), axis.line.y = element_line(color = "black",
size = .5), axis.ticks=element_line(size=2),
      axis.title.y=element_text(size=22), axis.title.x=element_text(size=0),
      axis.text.x=element_text(angle=35, hjust=1,size=18, colour="black"),
axis.text.y=element_text(size=18, colour="black"),
      panel.grid.minor=element_blank(), panel.grid.major=element_blank(),
      legend.title=element_blank())
print(graph.dataset.dd.manip.hab)
ggsave("D:/Jerem/Documents/Manip Lyon/perceptif
changeant/graph_dataset_dd_manipIduCldu_2mois_habituation.svg")


##### IDU CLDU
dataset.densite.idu.cldu <- read.csv("densite_idu_cldu.csv", header=T, sep=",")

dataset.densite.idu <- dataset.densite.idu.cldu[dataset.densite.idu.cldu$marqueur=="idu", ]
dataset.densite.cldu <- dataset.densite.idu.cldu[dataset.densite.idu.cldu$marqueur=="cldu", ]

dataset.densite.idu.lm <- lm(densite~groupe, data=dataset.densite.idu)
anova(dataset.densite.idu.lm)
print(summary(glht(dataset.densite.idu.lm,linfct=mcp(groupe="Tukey"))))

dataset.densite.cldu.lm <- lm(densite~groupe, data=dataset.densite.cldu)
anova(dataset.densite.cldu.lm)
print(summary(glht(dataset.densite.cldu.lm,linfct=mcp(groupe="Tukey"))))



infodata.densite.idu <- ddply(dataset.densite.idu,c("groupe"),summarise,
                       N = length(densite), mean=mean(densite), sd=sd(densite), sem=sd/sqrt(N))

infodata.densite.idu$groupe <- factor(infodata.densite.idu$groupe, levels=c("groupe 1", "groupe 2",
"groupe 3", "groupe 4"))
dataset.densite.idu$groupe <- factor(dataset.densite.idu$groupe, levels=c("groupe 1", "groupe 2",
"groupe 3", "groupe 4"))

graph.dataset.densite.idu<-ggplot((data=infodata.densite.idu),aes(x=groupe,y=mean, fill=groupe)) +
  geom_jitter(data = dataset.densite.idu,
```

```
            aes(x=groupe,y=densite),size=1.5, show.legend = FALSE,
            position = position_jitterdodge(jitter.width = 0.25, jitter.height = 0, dodge.width = 0.6)) +
  geom_bar(stat="identity", show.legend=FALSE, position=position_dodge(width=0.6), colour =
"black", width=0.6, alpha=0.7)+
  geom_errorbar(aes(ymin=mean,ymax=mean+sem, width=0.3),
position=position_dodge(width=0.6))+
  scale_x_discrete(labels = c("Group 1", "Group 2", "Group 3", "Group 4"))+
  scale_fill_manual(values=c("grey90", "grey70", "grey50", "grey30"))+
  scale_y_continuous("Idu-positive cell density (cell/?m?)",
                  limits=c(-0.001,8), breaks=c(0,1,2,3,4,5,6,7,8),
                  expand = c(0,0))+
  labs(title="")+
  theme(panel.background=element_rect(fill="white"),plot.title=element_text(size=18),
       axis.line.x=element_line(color = "black", size= .5), axis.line.y = element_line(color = "black",
size = .5), axis.ticks=element_line(size=2),
       axis.title.y=element_text(size=22), axis.title.x=element_text(size=0),
       axis.text.x=element_text(angle=0, hjust=0.5,size=18, colour="black"),
axis.text.y=element_text(size=18, colour="black"),
       panel.grid.minor=element_blank(), panel.grid.major=element_blank(),
       legend.title=element_blank())
print(graph.dataset.densite.idu)
ggsave("D:/Jerem/Documents/Manip Lyon/perceptif changeant/graph_dataset_idu_2mois.svg",
width=7, height=8)


infodata.densite.cldu <- ddply(dataset.densite.cldu,c("groupe"),summarise,
                         N = length(densite), mean=mean(densite), sd=sd(densite), sem=sd/sqrt(N))

infodata.densite.cldu$groupe <- factor(infodata.densite.cldu$groupe, levels=c("groupe 1", "groupe
2", "groupe 3", "groupe 4"))
dataset.densite.cldu$groupe <- factor(dataset.densite.cldu$groupe, levels=c("groupe 1", "groupe 2",
"groupe 3", "groupe 4"))

graph.dataset.densite.cldu<-ggplot((data=infodata.densite.cldu),aes(x=groupe,y=mean,
fill=groupe)) +
  geom_jitter(data = dataset.densite.cldu,
            aes(x=groupe,y=densite),size=1.5, show.legend = FALSE,
            position = position_jitterdodge(jitter.width = 0.25, jitter.height = 0, dodge.width = 0.6)) +
  geom_bar(stat="identity", show.legend=FALSE, position=position_dodge(width=0.6), colour =
"black", width=0.6, alpha=0.7)+
  geom_errorbar(aes(ymin=mean,ymax=mean+sem, width=0.3),
position=position_dodge(width=0.6))+
  scale_x_discrete(labels = c("Group 1", "Group 2", "Group 3", "Group 4"))+
  scale_fill_manual(values=c("grey90", "grey70", "grey50", "grey30"))+
  scale_y_continuous("cldu-positive cell density (cell/?m?)",
                  limits=c(-0.001,8), breaks=c(0,1,2,3,4,5,6,7,8),
                  expand = c(0,0))+
  labs(title="")+
  theme(panel.background=element_rect(fill="white"),plot.title=element_text(size=18),
       axis.line.x=element_line(color = "black", size= .5), axis.line.y = element_line(color = "black",
size = .5), axis.ticks=element_line(size=2),
       axis.title.y=element_text(size=22), axis.title.x=element_text(size=0),
```

```
        axis.text.x=element_text(angle=0, hjust=0.5,size=18, colour="black"),
axis.text.y=element_text(size=18, colour="black"),
        panel.grid.minor=element_blank(), panel.grid.major=element_blank(),
        legend.title=element_blank())
print(graph.dataset.densite.cldu)
ggsave("D:/Jerem/Documents/Manip Lyon/perceptif changeant/graph_dataset_cldu_2mois.svg",
width=7, height=8)




#############################
##### FIGURE 4 ############
##########################

rm(list = ls());

#Pachages----
library(reshape2)
library(ggplot2)
library(plyr)
library(nlme)
library(multcomp)
library(openxlsx)

#Reading dataset----
setwd("/media/jeremy/Data/CloudStation/temporaire/papier changeant/data_csv/matrice
behavior_whole_afterremoving_hab1=0_novirus2.xlsx")
setwd("N:/neuropop/J?r?my/J?r?my/Recherches/Th?se + Stage M2/Recherches/Manip
apprentissage perceptif changeant/Opto/T2 prime Group3 timing cldu Opto haloR")

dataset<-read.xlsx("matrice behavior_whole_afterremoving_hab1=0_novirus2.xlsx", "Feuil1")
dataset<-dataset[,(1:11)]
dataset<-na.omit(dataset)
dataset<-subset(dataset, hab1>1, select=souris:odorant)
dataset2<-melt(dataset,
measure.vars=c("mo","hab1","hab2","hab3","hab4","test"),factorAsStrings=F)
names(dataset2)[7]<-"temps"
names(dataset2)[6]<-"session"

attach(dataset2)


dataset2<-subset(dataset2, session!="mo", select=souris:temps)

#graphiques:

dataset2.lim <- dataset2[dataset2$odorant=="lim" , ]
dataset2.lim <- na.omit(dataset2.lim)

dataset2.dd <- dataset2[dataset2$odorant=="dd", ]
dataset2.dd <- na.omit(dataset2.dd)
```

```
dataset2.lcarv <- dataset2[dataset2$odorant=="lcarv", ]
dataset2.lcarv <- na.omit(dataset2.lcarv)

dataset2.lim <- dataset2.lim[dataset2.lim$laser != "off", ]

#####################################################################################
##########################
############################################     graphiques
############################################
#####################################################################################
##########################

### LIMONENE
infodata.dataset2.lim <- ddply(dataset2.lim,c("groupe", "session"),summarise,
                         N = length(temps), mean=mean(temps), sd=sd(temps), sem=sd/sqrt(N))

graph.dataset2.lim<-ggplot((data=infodata.dataset2.lim), aes(x=session,y=mean, fill=groupe)) +
  geom_jitter(data = dataset2.lim, aes(x=session,y=temps, fill=groupe),size=1.5,
            show.legend = FALSE, position = position_jitterdodge(jitter.width = 0.25, jitter.height = 0,
dodge.width = 0.6)) +
  geom_bar(stat="identity", show.legend=TRUE, position=position_dodge(width=0.6), colour =
"black", width=0.6, alpha=0.7)+
  geom_errorbar(aes(ymin=mean,ymax=mean+sem, width=0.3),
position=position_dodge(width=0.6))+
  scale_x_discrete(labels = c("Hab 1", "Hab 2", "Hab 3", "Hab 4", "Test"))+
  scale_fill_manual(values=c("grey90","grey30"))+
  scale_y_continuous("Investigation time (s)",
                limits=c(-0.01,8), breaks=c(0,1,2,3,4,5,6,7),
                expand = c(0,0))+
  labs(title="(+)limonene / (-)limonene")+
  theme(panel.background=element_rect(fill="white"),plot.title=element_text(size=18),
       axis.line.x=element_line(color = "black", size= .5), axis.line.y = element_line(color = "black",
size = .5), axis.ticks=element_line(size=2),
       axis.title.y=element_text(size=24), axis.title.x=element_text(size=0),
       axis.text.x=element_text(size=20, colour="black"), axis.text.y=element_text(size=20,
colour="black"),
       panel.grid.minor=element_blank(), panel.grid.major=element_blank(),
       legend.title=element_blank())
print(graph.dataset2.lim)
ggsave("N:/neuropop/J?r?my/J?r?my/Recherches/Th?se + Stage M2/Recherches/Manip
apprentissage perceptif changeant/Opto/T2 prime Group3 timing cldu Opto
haloR/graph_dataset_opto_lim.svg", width=8, height=13)
ggsave("D:/Jerem/Documents/Manip Lyon/perceptif changeant/opto/graph_dataset_opto_lim.svg")

### DD
infodata.dataset2.dd <- ddply(dataset2.dd,c("groupe", "session"),summarise,
                        N = length(temps), mean=mean(temps), sd=sd(temps), sem=sd/sqrt(N))

graph.dataset2.dd<-ggplot((data=infodata.dataset2.dd), aes(x=session,y=mean, fill=groupe)) +
  geom_jitter(data = dataset2.dd, aes(x=session,y=temps, fill=groupe),size=1.5,
```

```
            show.legend = FALSE, position = position_jitterdodge(jitter.width = 0.25, jitter.height = 0, dodge.width = 0.6)) +
  geom_bar(stat="identity", show.legend=TRUE, position=position_dodge(width=0.6), colour = "black", width=0.6, alpha=0.7)+
  geom_errorbar(aes(ymin=mean,ymax=mean+sem, width=0.3), position=position_dodge(width=0.6))+
  scale_x_discrete(labels = c("Hab 1", "Hab 2", "Hab 3", "Hab 4", "Test"))+
  scale_fill_manual(values=c("grey90","grey30"))+
  scale_y_continuous("Investigation time (s)",
                 limits=c(-0.01,8), breaks=c(0,1,2,3,4,5,6,7),
                 expand = c(0,0))+
  labs(title="decanal / dodecanone")+
  theme(panel.background=element_rect(fill="white"),plot.title=element_text(size=18),
        axis.line.x=element_line(color = "black", size= .5), axis.line.y = element_line(color = "black", size = .5), axis.ticks=element_line(size=2),
        axis.title.y=element_text(size=24), axis.title.x=element_text(size=0),
        axis.text.x=element_text(size=20, colour="black"), axis.text.y=element_text(size=20, colour="black"),
        panel.grid.minor=element_blank(), panel.grid.major=element_blank(),
        legend.title=element_blank())
print(graph.dataset2.dd)
ggsave("N:/neuropop/J?r?my/J?r?my/Recherches/Th?se + Stage M2/Recherches/Manip apprentissage perceptif changeant/Opto/T2 prime Group3 timing cldu Opto haloR/graph_dataset_opto_dd.svg", width=8, height=13)
ggsave("D:/Jerem/Documents/Manip Lyon/perceptif changeant/opto/graph_dataset_opto_dd.svg")

### Limonene+ Carvone
infodata.dataset2.lcarv <- ddply(dataset2.lcarv,c("groupe", "session"),summarise,
                         N = length(temps), mean=mean(temps), sd=sd(temps), sem=sd/sqrt(N))

graph.dataset2.lcarv<-ggplot((data=infodata.dataset2.lcarv), aes(x=session,y=mean, fill=groupe)) +
  geom_jitter(data = dataset2.lcarv, aes(x=session,y=temps, fill=groupe),size=1.5,
            show.legend = FALSE, position = position_jitterdodge(jitter.width = 0.25, jitter.height = 0, dodge.width = 0.6)) +
  geom_bar(stat="identity", show.legend=TRUE, position=position_dodge(width=0.6), colour = "black", width=0.6, alpha=0.7)+
  geom_errorbar(aes(ymin=mean,ymax=mean+sem, width=0.3), position=position_dodge(width=0.6))+
  scale_x_discrete(labels = c("Hab 1", "Hab 2", "Hab 3", "Hab 4", "Test"))+
  scale_fill_manual(values=c("grey90","grey30"))+
  scale_y_continuous("Investigation time (s)",
                 limits=c(-0.01,11), breaks=c(0,1,2,3,4,5,6,7,8,9,10,11),
                 expand = c(0,0))+
  labs(title="(+)limonene / (+)carvone")+
  theme(panel.background=element_rect(fill="white"),plot.title=element_text(size=18),
        axis.line.x=element_line(color = "black", size= .5), axis.line.y = element_line(color = "black", size = .5), axis.ticks=element_line(size=2),
        axis.title.y=element_text(size=24), axis.title.x=element_text(size=0),
        axis.text.x=element_text(size=20, colour="black"), axis.text.y=element_text(size=20, colour="black"),
        panel.grid.minor=element_blank(), panel.grid.major=element_blank(),
        legend.title=element_blank())
```

```
print(graph.dataset2.lcarv)
ggsave("N:/neuropop/J?r?my/J?r?my/Recherches/Th?se + Stage M2/Recherches/Manip
apprentissage perceptif changeant/Opto/T2 prime Group3 timing cldu Opto
haloR/graph_dataset_opto_lcarv.svg", width=8, height=13)
ggsave("D:/Jerem/Documents/Manip Lyon/perceptif
changeant/opto/graph_dataset_opto_lcarv.svg")

##################################################################################
#########################
##########################################     statistiques
##########################################
##################################################################################
#########################
ODORANT <- c("lim", "dd", "lcarv")
#LASER <- c("on", "off")
GROUPE <- c("cont", "halo")

for (odorant in ODORANT){
  for (groupe in GROUPE){
    print(paste0("Resultats pour ", groupe, " ", odorant))
    dataset2.hab <- dataset2[((dataset2$groupe == groupe & dataset2$odorant==odorant) &
(dataset2$session=="hab1"|
                                                            dataset2$session=="hab2"|
                                                            dataset2$session=="hab3"|
                                                            dataset2$session=="hab4")),]
    if (nrow(dataset2.hab) == 0) next
    else {
      dataset2.hab <- droplevels(dataset2.hab)
      dataset2.hab.lme <- lme(temps~session,random=~1|souris, data=dataset2.hab)
      print(anova(dataset2.hab.lme))
      #print(summary(glht(dataset2.hab.lme,linfct=mcp(session="Tukey"))))

      dataset2.discri <- dataset2[((dataset2$groupe == groupe & dataset2$odorant==odorant) &
(dataset2$session=="hab4"|
                                                               dataset2$session=="test")),]
      if (nrow(dataset2.discri) == 0) next
      else {
        var.test(dataset2.discri[dataset2.discri$session=='hab4',]$temps,
                 dataset2.discri[dataset2.discri$session=='test',]$temps)
        print(t.test(dataset2.discri[dataset2.discri$session=='hab4',]$temps,
                     dataset2.discri[dataset2.discri$session=='test',]$temps,
                     alternative = c("less"), var.equal = TRUE, paired = TRUE))
        readline(prompt="Press enter to continue")
      }
    }
  }
}
```

```
##########################################
######## ANALYSE CELLULAIRE ##############
########################################
dataset.cellulaire <- read.xlsx(file = "D:/Jerem/Documents/Manip Lyon/perceptif
changeant/opto/Photos YFP - Zif268/comptage2.xlsx",
                          "pour_R")

t.test(dataset.cellulaire[dataset.cellulaire$groupe=="cont", ]$gfp,
dataset.cellulaire[dataset.cellulaire$groupe=="halo", ]$gfp,
       var.equal = TRUE)
t.test(dataset.cellulaire[dataset.cellulaire$groupe=="cont", ]$gfpZif,
dataset.cellulaire[dataset.cellulaire$groupe=="halo", ]$gfpZif,
       var.equal = TRUE, alternative = c("greater"))
t.test(dataset.cellulaire[dataset.cellulaire$groupe=="cont", ]$zifTotal,
dataset.cellulaire[dataset.cellulaire$groupe=="halo", ]$zifTotal,
       var.equal = TRUE, alternative = c("greater"))
t.test(dataset.cellulaire[dataset.cellulaire$groupe=="cont", ]$percentGfpZif,
dataset.cellulaire[dataset.cellulaire$groupe=="halo", ]$percentGfpZif,
       var.equal = TRUE, alternative = c("greater"))



#################################################
############## GRAPHIQUES ######################
#################################################

infodata.dataset.cellulaire.gfp <- ddply(dataset.cellulaire,c("groupe"),summarise,
                                N = length(gfp), mean=mean(gfp), sd=sd(gfp), sem=sd/sqrt(N))

graph.dataset.cellulaire.gfp<-ggplot((data=infodata.dataset.cellulaire.gfp), aes(x=groupe,y=mean,
fill=groupe)) +
  geom_jitter(data = dataset.cellulaire, aes(x=groupe,y=gfp, fill=groupe),size=1.5,
            show.legend = FALSE, position = position_jitterdodge(jitter.width = 0.25, jitter.height = 0,
dodge.width = 0.6)) +
  geom_bar(stat="identity", show.legend=FALSE, position=position_dodge(width=0.6), colour =
"black", width=0.6, alpha=0.7)+
  geom_errorbar(aes(ymin=mean,ymax=mean+sem, width=0.3),
position=position_dodge(width=0.6))+
  scale_x_discrete(labels = c("Control", "Halorhodospin"))+
  scale_fill_manual(values=c("grey90","grey30"))+
  scale_y_continuous("GFP-positive cell density (cell/um?)",
                  limits=c(-0.0000001,0.0002), breaks=c(0,0.0001,0.0002),
                  expand = c(0,0))+
  labs(title="")+
  theme(panel.background=element_rect(fill="white"),plot.title=element_text(size=18),
        axis.line.x=element_line(color = "black", size= .5), axis.line.y = element_line(color = "black",
size = .5), axis.ticks=element_line(size=2),
        axis.title.y=element_text(size=22), axis.title.x=element_text(size=0),
        axis.text.x=element_text(size=18, colour="black"), axis.text.y=element_text(size=18,
colour="black"),
        panel.grid.minor=element_blank(), panel.grid.major=element_blank(),
        legend.title=element_blank())
```

```
print(graph.dataset.cellulaire.gfp)
ggsave("D:/Jerem/Documents/Manip Lyon/perceptif changeant/graph_dataset_cellulaire_gfp.svg")



dataset.cellulaire <- na.omit(dataset.cellulaire)
infodata.dataset.cellulaire.zifTotal <- ddply(dataset.cellulaire,c("groupe"),summarise,
                                   N = length(zifTotal), mean=mean(zifTotal), sd=sd(zifTotal),
sem=sd/sqrt(N))

graph.dataset.cellulaire.zifTotal<-ggplot((data=infodata.dataset.cellulaire.zifTotal),
aes(x=groupe,y=mean, fill=groupe)) +
  geom_jitter(data = dataset.cellulaire, aes(x=groupe,y=zifTotal, fill=groupe),size=1.5,
            show.legend = FALSE, position = position_jitterdodge(jitter.width = 0.25, jitter.height = 0,
dodge.width = 0.6)) +
  geom_bar(stat="identity", show.legend=FALSE, position=position_dodge(width=0.6), colour =
"black", width=0.6, alpha=0.7)+
  geom_errorbar(aes(ymin=mean,ymax=mean+sem, width=0.3),
position=position_dodge(width=0.6))+
  scale_x_discrete(labels = c("Control", "Halorhodospin"))+
  scale_fill_manual(values=c("grey90","grey30"))+
  scale_y_continuous("Zif268-positive cell density (cell/um?)",
                  limits=c(-0.000001,0.003), breaks=c(0,0.001,0.002, 0.003),
                  expand = c(0,0))+
  labs(title="")+
  theme(panel.background=element_rect(fill="white"),plot.title=element_text(size=18),
      axis.line.x=element_line(color = "black", size= .5), axis.line.y = element_line(color = "black",
size = .5), axis.ticks=element_line(size=2),
      axis.title.y=element_text(size=22), axis.title.x=element_text(size=0),
      axis.text.x=element_text(size=18, colour="black"), axis.text.y=element_text(size=18,
colour="black"),
      panel.grid.minor=element_blank(), panel.grid.major=element_blank(),
      legend.title=element_blank())
print(graph.dataset.cellulaire.zifTotal)
ggsave("D:/Jerem/Documents/Manip Lyon/perceptif
changeant/graph_dataset_cellulaire_zifTotal.svg")




infodata.dataset.cellulaire.percentGfpZif <- ddply(dataset.cellulaire,c("groupe"),summarise,
                                     N = length(percentGfpZif), mean=mean(percentGfpZif),
sd=sd(percentGfpZif), sem=sd/sqrt(N))

graph.dataset.cellulaire.percentGfpZif<-ggplot((data=infodata.dataset.cellulaire.percentGfpZif),
aes(x=groupe,y=mean, fill=groupe)) +
  geom_jitter(data = dataset.cellulaire, aes(x=groupe,y=percentGfpZif, fill=groupe),size=1.5,
            show.legend = FALSE, position = position_jitterdodge(jitter.width = 0.25, jitter.height = 0,
dodge.width = 0.6)) +
  geom_bar(stat="identity", show.legend=FALSE, position=position_dodge(width=0.6), colour =
"black", width=0.6, alpha=0.7)+
```

```
  geom_errorbar(aes(ymin=mean,ymax=mean+sem, width=0.3),
position=position_dodge(width=0.6))+
  scale_x_discrete(labels = c("Control", "Halorhodospin"))+
  scale_fill_manual(values=c("grey90","grey30"))+
  scale_y_continuous("GFP-positive / Zif268-positive cell (%)",
                     limits=c(-0.01,60), breaks=c(0,20,40,60),
                     expand = c(0,0))+
  labs(title="")+
  theme(panel.background=element_rect(fill="white"),plot.title=element_text(size=18),
        axis.line.x=element_line(color = "black", size= .5), axis.line.y = element_line(color = "black",
size = .5), axis.ticks=element_line(size=2),
        axis.title.y=element_text(size=22), axis.title.x=element_text(size=0),
        axis.text.x=element_text(size=18, colour="black"), axis.text.y=element_text(size=18,
colour="black"),
        panel.grid.minor=element_blank(), panel.grid.major=element_blank(),
        legend.title=element_blank())
print(graph.dataset.cellulaire.percentGfpZif)
ggsave("D:/Jerem/Documents/Manip Lyon/perceptif
changeant/graph_dataset_cellulaire_percentGfpZif.svg")
```
